# Tracing the Century‐Long Evolution of Microplastics Deposition in a Cold Seep

**DOI:** 10.1002/advs.202206120

**Published:** 2023-02-03

**Authors:** Jing‐Chun Feng, Can‐Rong Li, Li Tang, Xiao‐Nan Wu, Yi Wang, Zhifeng Yang, Weiyu Yuan, Liwei Sun, Weiqiang Hu, Si Zhang

**Affiliations:** ^1^ School of Ecology Environment and Resources Guangdong University of Technology Guangzhou 510006 P. R. China; ^2^ Southern Marine Science and Engineering Guangdong Laboratory (Guangzhou) Guangzhou 511458 P. R. China; ^3^ Guangdong Provincial Key Laboratory of Water Quality Improvement and Ecological Restoration for Watersheds Institute of Environmental and Ecological Engineering Guangdong University of Technology Guangzhou 510006 China; ^4^ Key Laboratory of Gas Hydrate Guangzhou Institute of Energy Conversion Chinese Academy of Sciences Guangzhou 510640 P. R. China; ^5^ Guangzhou Center for Gas Hydrate Research Chinese Academy of Sciences Guangzhou 510640 P. R. China; ^6^ South China Sea Institute of Oceanology Chinese Academy of Sciences Guangzhou 510301 P. R. China

**Keywords:** century‐long evolution, cold seep, methane, microplastics, natural gas hydrate

## Abstract

Microplastic (MP) pollution is one of the greatest threats to marine ecosystems. Cold seeps are characterized by methane‐rich fluid seepage fueling one of the richest ecosystems on the seafloor, and there are approximately more than 900 cold seeps globally. While the long‐term evolution of MPs in cold seeps remains unclear. Here, how MPs have been deposited in the Haima cold seep since the invention of plastics is demonstrated. It is found that the burial rates of MPs in the non‐seepage areas significantly increased since the massive global use of plastics in the 1930s, nevertheless, the burial rates and abundance of MPs in the methane seepage areas are much lower than the non‐seepage area of the cold seep, suggesting the degradation potential of MPs in cold seeps. More MP‐degrading microorganism populations and functional genes are discovered in methane seepage areas to support this discovery. It is further investigated that the upwelling fluid seepage facilitated the fragmentation and degradation behaviors of MPs. Risk assessment indicated that long‐term transport and transformation of MPs in the deeper sediments can reduce the potential environmental and ecological risks. The findings illuminated the need to determine fundamental strategies for sustainable marine plastic pollution mitigation in the natural deep‐sea environments.

## Graphical abstract

Century‐long evolution of microplastic deposition in cold seep sediment is investigated in detail. Microplastics in the non‐seepage areas can reflect the historical usage of synthetic polymers, while that in the methane seepage locations are more likely to be carried into the water column, or fragmented and degraded in the sediment.

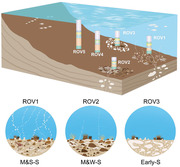

## 1 Introduction

Deep‐sea microplastic (MP) pollution constitutes a severe threat to sustainable and resilient oceans, as the deep sea is regarded as a> hotspot for MP deposition and accumulation.^[^ ^1^, ^2^, ^3^ ^]^ Once MPs enter the deep oceans, the heat transfer characteristics and porosity of sediments can be affected by MP accumulation, inducing alteration in benthic habitats.^[^ ^4^ ^]^ MPs can also function as a proxy for the transport of other pollutants, such as heavy metals, persistent organic pollutants, and environmental hormones, from the marine surface to the bottom, increasing the complex deep‐sea pollution phenomenon.^[^ ^5^ ^]^ In addition, small‐scale MPs can be easily ingested by marine animals, causing respiratory disease and digestion problems. Contaminated fishes can also transfer MPs to humans through the food web.^[^ ^6^ ^]^

Since the first production of plastics in 1907,^[^ ^7^ ^]^ increasing amounts of plastic waste have been poured into the oceans and they can be degraded into MPs. Subsequently, these MPs settled onto the seafloor through various vectors, such as riverine inputs, sewage system discharge, atmospheric transportation, offshore activity generation, and in situ degradation of large plastic debris.^[^ ^3^, ^8^, ^9^, ^10^ ^]^ It was estimated that approximately 0.15−0.39 million tons of plastic debris could finally enter the global oceans within one year due to the massive use of personal protective equipment since the COVID‐19 pandemic.^[^ ^11^ ^]^ Coastline areas and seawater are affected by plastic pollution comprising gloves and face masks due to the COVID‐19 pandemic,^[^ ^12^ ^]^ and the influence of MPs in remote sea areas under the ongoing COVID‐19 pandemic should not be overlooked.^[^ ^13^ ^]^ Currently, MPs are widely discovered in the marine multimedia environment of water columns, sediment zone, and various organisms.

To uncover the harmfulness and risks of deep‐sea MPs, it is crucial to explore all possible deep‐sea MP sinks and hotspots worldwide.^[^ ^14^ ^]^ MP deposition on the seafloor is caused by more factors other than the simple passing through the water column, and the distribution is affected and reworked by seafloor fluid flows.^[^ ^3^ ^]^ Previous deep‐sea studies mainly concentrated on MPs originating from the continental shelf, continental slope, and abyss.^[^ ^15^ ^]^ It has been verified that the negative topography in oceanic trenches and submarine canyons is conducive to MP enrichment under the effects of gravity flows and oceanic currents.^[^ ^1^, ^16^ ^]^ However, little is known regarding the occurrence characteristics of MPs in positive topographies of hydrocarbon seeps. Supported by subsurface hydrocarbon reservoirs, commonly associated with natural gas or gas hydrate, hydrocarbon seeps are special deep‐sea benthic habitats where methane, hydrogen sulfide, and other reduced chemicals are released from the seafloor.^[^ ^17^ ^]^ Cold seeps are typical hydrocarbon seeps, where plumes carrying methane gases and sediment particles from the sea bed to the water column create complex turbulent and wake flow atmospheres in the seepage area.^[^ ^18^ ^]^ The seeping chemical compounds can be metabolized by the local micrograms, forming the base of the unique chemosynthetic communities with the absence of photosynthesis and sunlight.^[^ ^19^ ^]^ There are approximately 900 active cold seeps around the world, and strong plumes of methane bubble flow can reach as high as several hundred meters or one kilometer^[^ ^20^ ^]^ or can even be transported to the sea surface.^[^ ^21^ ^]^ The upwelling fluid flow influences MP sinks from seawater to the sediment, and microbial‐mediated methane oxidation processes in cold seeps function as large biological methane and carbon sinks in the dark world.^[^ ^22^, ^23^ ^]^ Cold seeps are always characterized by biological productivity, and the special communities that depend on chemosynthesis are regarded as biological oases in the normally nutrition‐poor deep sea.^[^ ^24^, ^25^ ^]^ These natural laboratories of rich carbon biotransformation may also influence the degradation of carbon‐based MPs, because plastics are petroleum‐based, and cold seeps are generally associated with natural gas and oil deposits, which are currently less understood. The differences in buried MPs between fluid seepage and non‐seepage areas could shed light on the effects and responses of the internal deep Earth environment regarding MP sequestration, and the characteristics of the development stage of cold seep are shown in Figure S1, Supporting Information.

The sustainable management of marine plastic pollution control depends on tracing the degradation characteristics and ultimate fate of various MPs in the deep sea on a long time scale. Although the deep seafloor is regarded as containing pervasive MPs, long‐term evolution analysis of MPs in the sediment is missing. Despite temporal evaluation studies of MPs in stratified sediment cores utilizing radioisotope methods widely conducted involving urban lake sediments,^[^ ^26^ ^]^ mangrove coastal areas,^[^ ^14^ ^]^ marine fishing bays,^[^ ^27^ ^]^ and shallow estuaries and bays,^[^ ^28^ ^]^ long‐term records of diffusion, fragmentation, and interaction of MPs in fluctuating and complex deep‐sea environments are lacking. This indispensable long‐term information can contribute to revealing plastic‐carbon effects on the deep lithosphere and biosphere.^[^ ^29^ ^]^ Only historical evaluation of MP deposition can prevent over‐ or underestimation of the environmental risks of MPs in the deep ocean,^[^ ^30^ ^]^ which is insufficient to date.

Different types and sizes of MPs contribute to various environmental hazards and health risks among marine organisms.^[^ ^31^ ^]^ Once MPs are deposited in the marine environment, they can be fragmented and degraded^[^ ^32^ ^]^ under the effects of current flows, biofouling, and ultraviolet exposure.^[^ ^33^ ^]^ It is difficult to identify MPs smaller than 63 µm, and these small‐scale MPs are more likely to be ingested by marine organisms, causing higher ecological risks.^[^ ^34^ ^]^ Hence, the neglect of small‐scale MPs causes underestimation of MPs occurring on the seafloor, and reliable technologies are needed to detect a wide range of MP sizes (0–5000 µm).

**Figure 1:**
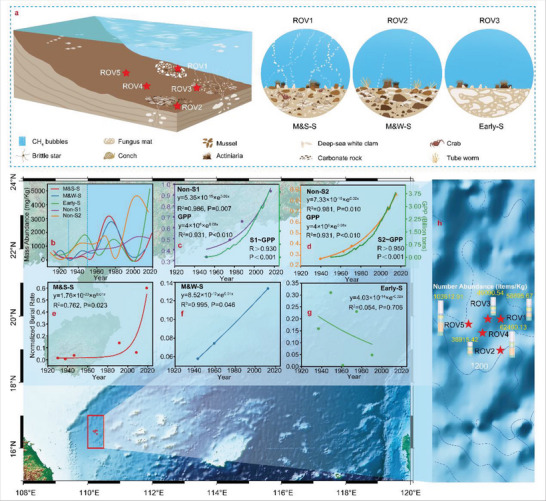
Geological information of the sampling locations and evolution of the abundance of the MPs. a) The geological and habitat distributions of the five sampling locations. b) Historical record of the MP mass abundance. c,d) Normalized MP burial rates in the non‐seepage areas and the amount of global plastic production. e–g) Normalized MP burial rates in the methane seepage areas. h) Total abundance with the spatial distribution.

To address the above problems, we detected and characterized, for the first time, the MP occurrence and degradation in cold seepage areas on a long‐time scale in this work. We utilized our patented technology^[^ ^35^, ^36^ ^]^ to identify a wide range of MP sizes (20–5000 µm) in sediment cores obtained in the Haima cold seep (**Figure** **1**a) to uncover the evolutions of MPs deposition in the methane seepage and non‐seepage areas. To better understand the key mechanism and driving factors of the ultimate fate of MPs in deep‐sea oceans at long‐term scales, the sediment dating is based on the age model of the ^210^Pb_bex_ activity decay and the peak value of ^137^Cs activity. We explored the role of the methane seepage level and other environmental factors, such as pore water geochemical factors and microorganism activity in sediments, on the rates of MP burial, fragmentation, and degradation. To achieve sustainable marine plastic use and disposal, we also analyzed the sources of MP generation and assessed the environmental risks of MPs on a centenary scale.

## 2 Results

### 2.1 Evaluation of Cold Seep Areas as Possible MP Sinks

A total of four typical habitat types of five diving locations were investigated. Seen in Figure 1, ROV1‐ROV5 denote the middle cold seep development stage with strong methane seepage (M&S‐S), the middle cold seep development stage with weak methane seepage (M&W ‐S), the early cold seep development stage (Early‐S), non‐seepage 1 stage (Non‐S1), and non‐seepage 2 stage (Non‐S2). Figure 1b shows that both at the onset of industrial plastic production in the 1930s^[^ ^37^ ^]^ and the massive production occurring in the 1950s,^[^ ^5^ ^]^ the mass abundance of MPs exhibited significant growth. MPs are common outcomes during the anthropic usage of plastic material and the ultimate fate of plastic waste in the environment. Land transportation and marine activities in coastal areas cause approximately 80% of plastic debris to enter the marine environment, and eventually, 99% of such plastic will end up in the deep ocean.^[^ ^1^, ^38^ ^]^ Therefore, as shown in Figure 1b, the historical trajectories of MPs deposited in the Haima cold seep could reflect the plastic production history. The MP burial rate in non‐seepage areas exponentially increased from the 1930s to the 2020s, which agrees with the exponential growth in the global plastic production rate (Figure 1c,d). The exponential growth tendency of the burial rate at the middle cold seep development stages was much weaker (Figure 1e,f), and the burial rate at the early stage decreased under fluctuations, suggesting that the MP accumulation rate in the sediment decreased with methane seepage. This occurred because the upwelling methane fluid flow could carry sediment particles into the overlying hydrosphere,^[^ ^39^ ^]^ and MPs may be utilized by active microorganism communities in active cold seep systems. The current burial rate (2001–2020) in the Haima cold seep (average: 248.73± 213.30 mg plastics m^−2^ year^−1^) is several times higher than that in coastal areas (64± 4 mg plastics m^−2^ year^−1^),^[^ ^14^ ^]^ suggesting that deep‐sea sediment functions as a much stronger MP sink. The total abundance of MPs is tens of thousands of items per kilogram of dry sediment, which is much higher than that in other marine environments.^[^ ^1^, ^2^ ^]^ This significant difference occurs because deep‐water zones provide a very high capacity to accommodate long‐term MP transportation, and the fine‐grained clay sediments in the Haima cold seep areas contribute to MP accumulation.^[^ ^40^ ^]^

**Figure 2:**
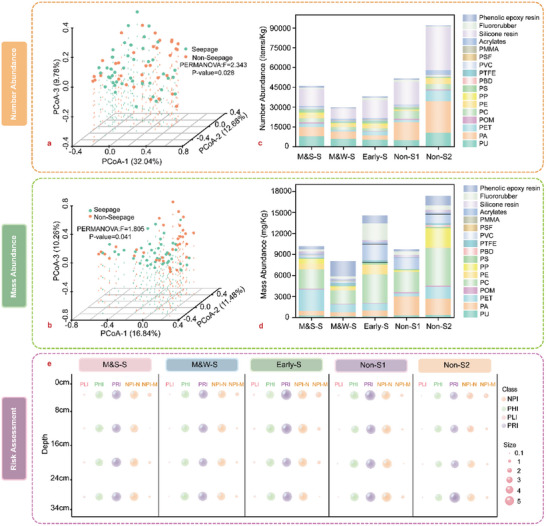
Polymer abundance and risk of MPs. a,b) Principal coordinate analysis (PCoA) based on the Bray Curtis dissimilarity metrics of the MP abundance between the methane seepage and non‐seepage areas. c,d) Number and mass abundance distributions of the 17 types of detected MPs. e) Environmental risk assessment of the different diving locations. Four indicators, the PLI, PRI, PHI, and NPI of the number abundance (NPI‐N), and Nemerow pollution index of the mass abundance (NPI‐M), are adopted to verify and calibrate the environmental harm caused by microplastics.^[^ ^41^, ^70^ ^]^ All of the risk indicators are normalized and divided into five categories.^[^ ^72^ ^]^

**Figure 3:**
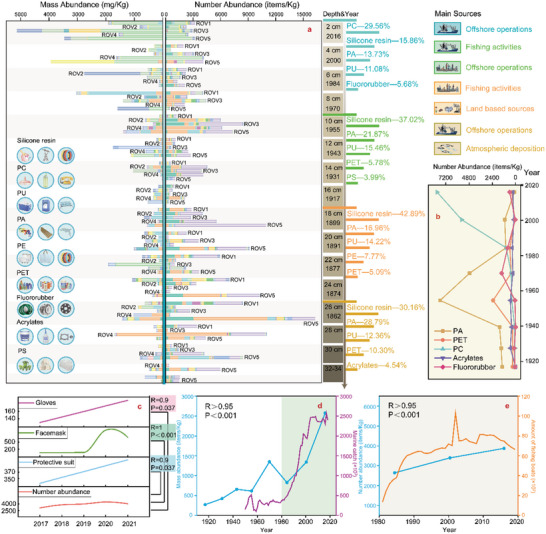
Source and sink characteristics of the different MP categories. a) The source analyses are based on the ingredients of the five major MPs within different time frames. Ingredient analysis is conducted by cutting the integral core into four subsections at 8 cm intervals. b) The temporal evolution of the abundance of the major five types of MPs. c) The relationship between the MP abundance and protective equipment used in recent years (data source: https://bg.qianzhan.com/). d,e) The relationship between the MP abundance and marine catches and fishing boats in the South China Sea. The statistical data originate from the China Social‐Economic Statistical Yearbook.

Principal coordinate analysis (PCoA)^[^ ^41^ ^]^ revealed that both the number and mass abundance exhibited a significant difference between the seepage and non‐seepage areas. The PERMANOVA *F* value for the number abundance (2.343) was higher than that for the mass abundance (1.805) of MPs, and the corresponding *P* value was much more significant (**Figure** **2**a,b). The number abundance of MPs in the non‐seepage areas was higher than that in the seepage areas (Figure 2c), and this result verified the previous conclusion that the accumulation ability of MPs in the methane seepage areas was lower. The number abundance of MPs at the M&S‐S stages was the highest among all the seepage locations because strong methane bubble flows were beneficial to particle accumulation near the seepage areas. The mass abundance varied among the different locations with a similar trend to that of the number abundance, except that the Early‐S stage exhibited the highest abundance among the seepage areas originating from large‐scale MPs with high densities of Polycarbonate (PC), Polyvinyl chloride (PVC), and Fluororubber (FKM) settling on the surface (**Figure** **3**a).

A comprehensive risk assessment was conducted to gain insight into the environmental risks of MPs based on the combined evaluation of four indicators (Figure 2e). The pollution load index (PLI) at all the diving locations was relatively low, while the polymer risk assessment index (PHI) and Nemerow pollution index (NPI) of the number abundance (NPI‐N) in this work were significantly high in all the areas and higher than those in sediments of other environments.^[^ ^42^ ^]^ This occurred because seventeen categories of MPs were detected at all five diving locations. The value of PLI in the Early‐S was the highest, while it decreased in the middle seepage stage. The potential ecological risk index (PRI) in the M&W‐S was also the lowest because of long‐term and weak methane seepage with fewer disturbances prior to MP degradation. For all the sampling sites, the contribution value of PC to PHI is the highest only in the surface layer, followed by Acrylates (AC) and Polyurethane (PU). The NPI‐N value in all the stations is relatively high, and it is higher in the non‐seepage areas. Furthermore, the NPI‐M value in the deeper layers decreased, suggesting that long‐term transport and transformation of MPs in the deeper sediments could reduce the potential environmental and ecological risks.

### 2.2 Evolution of the MP Sources Over Time

To trace the evolution of MP sources, we analyzed the changes in MP categories between stratigraphic decades. *R_N/M_*, the ratio of the number abundance (right side of Figure 3a) to the mass abundance (left side of Figure 3a), can be employed as an effective indicator to explain the fragmentation level of MPs. PC was primarily discovered in the upper 4 cm, and PC demonstrated obvious fragmentation with increasing *R_N/M_* value. Figure 3b also verifies that PC has only accumulated in recent years. Polyamide (PA) and Silicone resin (SI), which are widely used in engineering plastics and coatings in ships and marine engineering, significantly increased with the depth. These two MPs also exhibited a fragmented trend at sediment depths below 10 cm. *R_N/M_* of most MPs was higher in the methane seepage areas than that in the non‐seepage areas, indicating that the non‐seepage areas provided a higher capacity for the sequestration of large‐scale MPs. In the cold seepage areas, the upwelling methane fluid can lift denser and cold particles, and enable them to be entrained into the methane plume, which is beneficial for the particles, such as the MPs, to be carried into the water column,^[^ ^43^, ^44^ ^]^ resulting in a lower abundance of MPs in the sediment. In addition, the collision effect of methane bubbles and the strong fluid convection can enhance the interactions of the methane fluid and the surface of MPs, which could promote the fragmentation and aging process of MPs during transportation.^[^ ^36^ ^]^

In general, Figure 3a depicts that with the increase of sediment depth, engineering synthetic polymers such as PC associated with consumer goods declined, while chemical materials such as Polytetrafluoroethylene (PTFE) and AC applied to the chemical processing industry gradually increased. The overall evolution is mainly from light industrial plastic products on the top surface to heavy industrial plastic products in the deeper sediment. In the top 2 cm (approximately 2016–2021), the increase in the number abundance of MPs was closely correlated with the extensive use of virus transmissions prevention products, such as disposable protective clothing, safety gloves, and disposable masks, since the COVID‐19 pandemic (Figure 3c). In the deeper layers, the MP abundance was closely correlated with the number of fishing activities expressed as marine catches (Figure 3d) and the number of fishing boats (Figure 3e). Specifically, anthropic activities and offshore operations were the primary sources of MPs deposited in cold seepage environments.

### 2.3 Influence of Methane Seepage on MP Fragmentation and Degradation

**Figure 4:**
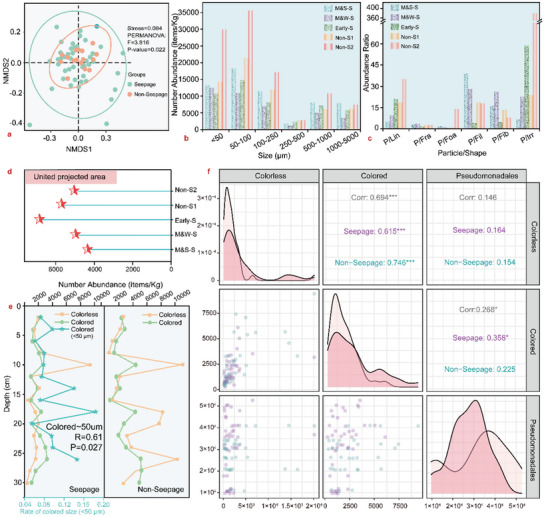
Morphology and degradation characteristics of MPs. a) Nonmetric multidimensional scaling test (NMDS) results of the size distribution of MPs among the methane seepage and non‐seepage areas. b) Size distribution of MPs. c) Abundance ratio of the particles to the other types. d) United projected area of MPs. e) Abundance of the colored and colorless MPs with depth. f) Correlation analysis of the colored and colorless MPs with the MP‐degrading *Pseudomonadales*.

*β* diversity based on the nonmetric multidimensional scaling (NMDS) method can denote the diversity difference among different samples,^[^ ^45^ ^]^ and **Figure** **4**a shows that the size distribution of MPs in the methane seepage and non‐seepage areas exhibited high *β* diversity, with a good result that the *P* value of the Permutational multivariate analysis of variance (PERMANOVA) testing method was only 0.022. This suggests that there was a significant difference in the size distribution of MPs among the methane seepage and non‐seepage areas. As seen in Figure 4b, small‐scale MPs (<250 µm) are significantly higher than that of the large‐scale MPs in all the sampling stations. The ratio of the particle abundance of small‐scale MPs to that of other morphologies can also reflect the fragmentation of MPs. Such indicator in the middle methane seepage areas is higher than that of the others for the dominant morphology of MPs with fragment, film, and fiber, (Figure 4c). In addition, Figure 4d also demonstrates that the projected areas of MPs in the methane seepage areas were smaller, verifying that the fragmentation degree responded more sensitively to methane seepage. The higher value at the Early‐S stage was attributable to newly sequestered PVC MPs at the surface. Moreover, Figure 4e indicates that along the sediment depth, the abundance of colored MPs synchronously varied with that of colorless MPs, and the strong correlation was verified in Figure 4f. Figure 4e also indicates that both the colored and colorless MP abundance levels peaked at depths of approximately 10 and 25 cm, which agreed well with the abundance peak of MP‐depredating microorganism *Pseudomonadales* in Figure S6, Supporting Information.^[^ ^46^ ^]^ It is also noted in Figure 4e that the abundance of fine‐scale MPs (<50 µm) relative to large‐scale colored MPs in these zones correspondingly increased, providing further evidence that the colored MPs were degraded and fragmented. In addition, Figure 4f shows that the microorganism level exhibited a positive correlation with colored MPs in the methane seepage areas, suggesting that MPs were more likely to be decomposed or degraded with methane seepage.

### 2.4 MP Abundance is Controlled by Geochemical and Microorganism Characteristics

**Figure 5:**
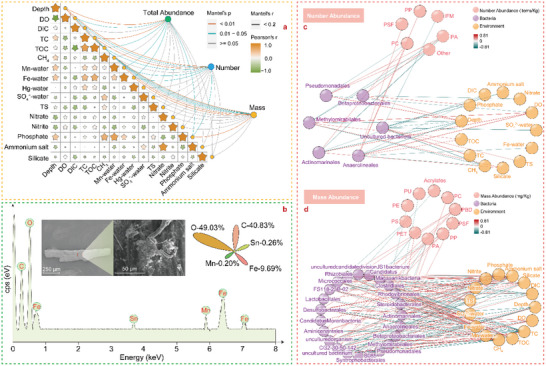
Environmental correlations with the MP abundance. a) Mantel tests results of the MP abundance and geochemical factors. b) SEM results of the MPs at ROV1. c) Network analysis of the abundance, geochemical factors, and microbial populations. d) Network analysis of the mass abundance, geochemical factors, and microbial populations. The microorganism population analyses are based on the operational taxonomic units (OTUs) of the bacterial 16S rRNA gene.

The MP abundance was controlled by environmental factors, as it was demonstrated that the depth and concentration of total organic carbon (TOC), total carbon (TC), iron (Fe) ions, manganese (Mn) ions, and phosphate in the sediment pore water were significantly correlated with the MP abundance based on Mantel's test method (**Figure** **5**a). Environmental factors were more closely correlated with the mass abundance than with the number abundance because these parameters could influence the MP degradation ability. The scanning electron microscopy‐energy dispersive spectroscopy (SEM‐EDS) test method indicated that Fe and Mn ions, which are electron acceptors of the anaerobic oxidation of methane (AOM) reaction, had accumulated on the surface of MPs^[^ ^47^ ^]^ (Figure 5b), providing indirect links between MPs and methane transformation. Network analysis revealed the relationship between the MP abundance, geochemical factors, and microorganism populations. There occurred a significant correlation between the number abundance of Polypropylene (PP), PA, Polysulfone (PSF), and PC and the population abundance of *Pseudomonadales*, *Betaproteobacteriales*, *Actinomarinales*, and certain uncultured bacteria. In addition, these microorganism populations were primarily correlated with the sediment depth and dissolved oxygen (DO), TOC, TC, silicate, and phosphate pore water concentrations (Figure 5c). This result suggested that these microorganisms could provide the potential to degrade the associated MPs. In addition, *Methylomirabilales* ^[^ ^48^ ^]^ indirectly affected MP degradation because they are closely linked with environmental factors influencing potential MP‐degrading microorganisms. Regarding the mass abundance, more types of polymers were affected by more microbial species, as the abundance of PC, PA, Polyethylene terephthalate (PET), PSF, PP, and PSF were significantly correlated with the microorganism abundance and concentration of environmental indicators (Figure 5d).

**Figure 6:**
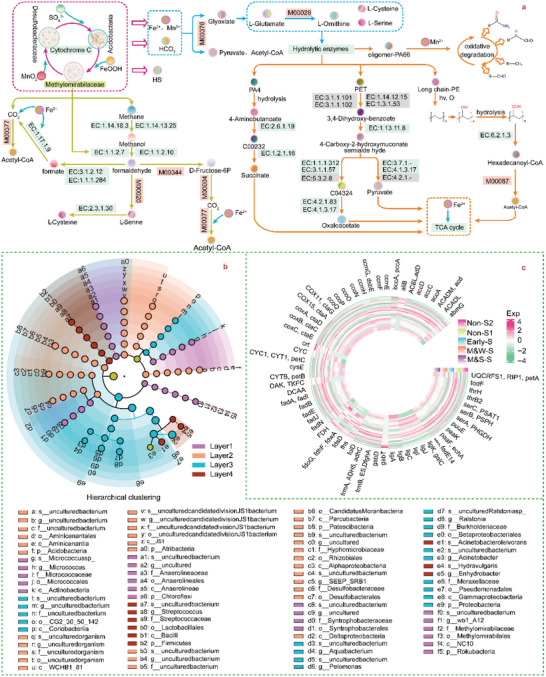
Biodegradation mechanism of MPs in the cold seepage area. a) Pathways of MP degradation and methane oxidation based on functional prediction from the KEGG Orthology database. The reported and undetected enzymes in the database are labeled green and gray, respectively. The module with the missing enzyme is marked in pink. b) Microorganism population distribution at the ROV locations with the sediment depth. c) Gene abundance distributions for alkane metabolism, MP monomer degradation, chemosynthesis, sediment carbon sequestration, Fe/Mn‐catalyzing enzyme, and electron transfer pathways in cytochromes among the different sampling locations. Functional prediction is based on the MicFunPred database with an average Spearman correlation coefficient of 0.89.

To further elucidate the biodegradation mechanism of MPs in the Haima cold seep environment, metabolic pathways of methane oxidation and MP degradation were proposed. As shown in **Figure** **6**a, in the AOM process, methane was stepwise transformed into methanol, formaldehyde, and formic acid and finally into serine and acetyl coenzyme A in the tricarboxylic acid cycle. Metallic minerals, sulfate‐reducing bacteria (SRB), and *Acidobacteria* were active in the Metal‐AOM process,^[^ ^49^ ^]^ in which SRB and *Acidobacteria* receive electrons released by methanotrophic bacteria when they oxidize methane and oxidize metal minerals through the “cryptic sulfur cycle”,^[^ ^50^ ^]^ releasing Fe^2+^, Mn^2+^, SO_4_^2−^, and S^2−^ in the whole process. The vector linking the extracellular electron transfer in the above process can be used by cytochrome C. Methanotroph has a large number of genes encoding cytochrome C, suggesting the importance of electron transfer in Metal‐AOM process. These substrates could enter the 3‐hydroxypropionic acid cycle, in which more glyoxylic acid, glutamic acid, and other amino acids were formed to promote the synthesis of extracellular PET‐degrading enzymes.^[^ ^51^ ^]^ According to the hydrolysis process of PET and biological metabolic path analysis, PET can first be hydrolyzed by extracellular degradation enzymes to produce small‐molecular weight Mono (ethyleneterephthalate). These hydrolyzed products are bioavailable and eventually can be promoted by dioxygenase to generate degradation products 3‐Carboxy‐cis,cis‐muconate^[^ ^52^ ^]^ and 4‐Carboxy‐2‐hydroxymuconate semialdehyde, which ultimately enter the metabolic pathway of benzoic acid. Considering the role of oxygen radicals and monooxygenase released by microflora in the sediments, oligomeric polyethylene (PE) could be degraded into a dimer and monomer primarily,^[^ ^53^ ^]^ and is eventually oxidized to low molecular weight substances.

To uncover the relationships between microorganisms and MP degradation, microorganism populations, and associated functional genes were analyzed in depth. In the strong methane seepage area, the microorganism populations significantly differed among the four vertical uniformly spaced zones, with a linear discrimination analysis (LDA) value larger than 4 (Figure S6, Supporting Information). As shown in Figure 6b, in the upper 8 cm, groups of *Syntrophobacterales*, *Anaerolineales*, and *Micrococcuss* species were dominant, and *Anaerolineales* could degrade PET. At a depth from 8–16 cm, the dominant population order was *JS1* > *Acidobacteria* > *Alphaproteobacteria*, in which *JS1* could degrade alkanes and synthetic polymers.^[^ ^54^ ^]^ At a depth of 16–24 cm, the dominant populations included *Pseudomonadales*, *Betaproteobacteriales*, and *Coriobacteriia*. Polymer‐degrading *Pseudomonadales* were the most dominant species. The bottom layer was primarily colonized by *Lactobacillales*, which exhibited the potential to biodegrade plastics,^[^ ^55^, ^56^ ^]^ and *Hydra vulgaris*, which produced a cellulolytic enzyme.^[^ ^57^ ^]^ Cellulolytic enzymes are composed of a variety of complex enzyme systems, which can break the glycosidic bond and gradually hydrolyze macromolecular cellulose into glucose. Most of them can degrade biodegradable plastics^[^ ^8^ ^]^ with the same active site, such as polylactic acid (PLA), polyhydroxyalkanoic acid (PHA), and polyvinyl alcohol mixed with starch. In summary, microorganisms in the strong methane seepage area could degrade plastics. We further compared the distribution of the functional genes among the different sampling locations (Figure 6c). Functional genes that potentially degrade monomers associated with synthetic polymers were discovered at all five sites, such as the PA4‐degrading gene (puuE|gabT|gabD), PE‐metabolizing gene (|paaK|ACSL, fadD|abmG|), and PET‐degrading gene (ligB|ligA|ligC|ligI|todF|ligK, galC|ligJ|). Other indirect genes promoting MP metabolism, as shown in Figure 6a, consisted of methane metabolism, metal electron transport, and carbon fixation genes, and they were also widely discovered. Figure 6c shows that the gene abundance for PA degradation in the methane seepage zones was higher than that in the non‐seepage zones. The early seepage area contained the most PE‐degrading genes. From the perspective of a metal‐driven AOM process, the methane seepage area contained abundant genes for metal electron transport and methane metabolism, which provided the potential to produce more indispensable serine, cysteine, and ornithine to promote the synthesis of extracellular MP‐degrading enzymes.

## 3 Discussion

The deep sea is widely considered the ultimate sink and hotspot for anthropic plastic pollution.^[^ ^1^ ^]^ A global seafloor MP estimation map does still not exist; however, the evaluation of MPs in unique ecosystems relying on chemosynthesis in the dark world is especially missing. It has been proposed to estimate how the Earth system may respond to long‐term MP production in detail. To reveal the persistent environmental risk of MPs, it must be understood how much MPs are annually buried in marine sediments and how MP‐derived carbon interacts with marine organic carbon.^[^ ^58^ ^]^ Unfortunately, little attention has been given to the long‐term assessment of MP occurrences in deep‐water sediments compared to that of MP occurrences in lakes^[^ ^26^ ^]^ and coastline areas.^[^ ^14^ ^]^

The extraction and identification process may fragment local MPs into smaller pieces, causing uncertainty in the evaluation of the number abundance of MPs. The mass abundance could be utilized as a more objective proxy to demonstrate the MP occurrence and a significant increase in MPs in sediments with the onset of industry and mass production in the 1930s and 1950s. This suggests that mass abundance could be utilized as a fingerprint of plastic pollution. We also discovered that the burial rate of MPs in the cold seepage areas was several times higher than that in coastal sediments. Uncaptured MPs in nearshore areas and the overlying water column could finally be deposited in deep‐sea sediments. In addition, the convolution flow attributed to methane seepage upwelling facilitated particle accumulation near the seepage points. Furthermore, large amounts of fine‐scale MPs (20–5000 µm) were identified via our established combined method. Long‐term MP identification in deep‐sea sediments could provide a timeline of MP sequestration and degradation, which is essential to elucidate the accurate environmental risks of MP dumping into the deep sea.

Historical records revealed that the cumulative MP burial rates in the cold seep with methane seepage sites were significantly lower than that without methane seepage locations, especially for the burial rates before 2000. In addition, colored MP fragmentation was more notably correlated with *Pseudomonadales* in the methane seepage zones, and the MP‐degrading function genes were more abundant in the methane seepage areas. This result suggested that methane fluid effusion could provide the potential to promote MP fragmentation and degradation. The cold seepage area provides a favorable environment for methanogenic and sulfate‐metabolizing bacteria. It has been shown that under anaerobic conditions, some synthetic polymers (e.g., additives, plasticizers, etc.) can be metabolized by methanogens or sulfate‐reducing agents to produce CH_4_ and CO_2_,^[^ ^59^ ^]^ respectively, demonstrating that the synthetic polymers accumulated in deep ocean bottom sediments are degraded, especially in methane seepage areas. This is attributed to the fact that it has more suitable conditions to accelerate the oxidation degradation of the polymer, such as continuous dense bubble flow, oxidation contact time, and degradation microorganisms. It has been reported that MP degradation in benthic systems could occur slower than that on the land surface because of a lack of UV radiation, lower temperatures, and oxygen deficiency.^[^ ^60^ ^]^ In this study, however, we discovered that high‐efficiency chemosynthesis in dark and cold systems could promote MP degradation.

The Mantel tests indicated that the methane concentration was not directly related to the MP abundance. However, the MP abundance was strongly affected by the reactant and product concentrations in the AOM process, which suggested that methane oxidation could indirectly influence MP degradation. The metabolic pathways further explained why the AOM process could promote the synthesis of extracellular MP‐degrading enzymes. Methane seepage in cold seeps entails a dynamic and intermittent process,^[^ ^61^ ^]^ and current methane concentration monitoring is restricted to one moment reflecting the imbalance in long‐term MP accumulation. Hence, long‐term methane flux monitoring may be directly correlated with MP degradation, and this must be verified in the near future.

The interaction between MPs and deep‐sea methane seepage processes is very important, as the majority of methane originates from methane hydrate dissociation. Learning from the natural mechanism of methane transformation to promote MP degradation could enlighten us to determine efficient synthetic polymer degradation strategies, as the deep‐sea methane transformation process could be simulated in a high‐pressure bioreactor.^[^ ^23^, ^62^, ^63^ ^]^ In addition, the response of methane transformation and transportation functions must be clarified, as the surface of aged MPs can accommodate large amounts of metal ions and microorganisms that can influence the AOM efficiency. Methane imposes a stronger greenhouse effect than that carbon dioxide (CO_2_), and the deep‐sea environments associated with natural gas hydrates could function as the largest global methane reservoir.^[^ ^64^ ^]^ Therefore, more attention is required to uncover the long‐term interactions between MPs and deep‐sea methane systems in pursuit of less MP pollution and lower greenhouse gas emissions.^[^ ^65^ ^]^

In contrast to small‐scale (<63 µm) MPs that cannot be identified via the single Fourier transform infrared spectroscopy (FTIR) test method,^[^ ^1^, ^33^ ^]^ we applied our patented method, which combined the test methods of visual observation, FTIR, and laser direct infrared imaging (LDIR). Fine‐scale MPs (20–500 µm) could be identified via the LDIR method after several flotation and dissolution cycles.^[^ ^66^ ^]^ Furthermore, we detected a total of 17 categories of MPs, which is far more than that detected in previous work.^[^ ^1^, ^27^, ^33^, ^58^ ^]^ This result verified the high sensitivity of our established method. Hence, this work provides a reference to assess full‐scale MP extraction and identification from sediments.

Effective indicators are essential to assess the ecological and environmental risks of MPs.^[^ ^67^ ^]^ To avoid bias, we applied four indicators to assess the MP risk among the different sampling locations. The PRI combines the pollution and polymer loads, and the NPI can effectively reduce the uncertainty in sampling locations and identification processes. Therefore, joint evaluation involving the PRI and NPI could be considered a holistic method to assess MP risks. In the future, in contrast to these static indicators, dynamic indicators, such as the correlation of the MP degradation rate, should also be considered to investigate the long‐term and dynamic MP risks.

Historical MP evaluations in the deep sea play an important role in clarifying the effects of anthropogenic activity on the inner depths of the Earth. Reducing the discharge of synthetic polymers into the oceans from land‐ and offshore‐based activities are required at the global, national, and regional management scales. The gradually degraded types of MPs in sediments provide new perspectives for sustainable offshore plastic use to replace un‐degraded categories. We could also take advantage of cold seep areas as natural laboratories to culture biodegradable microorganisms or use chemical degradation technologies to facilitate sustainable oceans by controlling plastic pollution.^[^ ^63^ ^]^

## 4 Conclusion

We have investigated the century‐long evolution of MPs deposition wide range of sizes (20–5000 µm) by our patented method in a cold seep of the deep sea. The current burial rate of MPs in the Haima cold seep is several times higher than that in coastal areas, suggesting that deep‐sea sediment functions as a much stronger MP sink. We demonstrated that the burial rates of MPs in the non‐seepage areas significantly increased since the massive global use of synthetic polymers in the 1930s, while the methane seepage areas have MPs potential of degradation for the detected lower burial rates and abundance from the long‐term scale. More MP‐degrading microorganism populations and functional genes were also discovered in methane seepage areas to support this discovery. We further investigated that the upwelling fluid seepage facilitated the fragmentation and degradation behaviors of MPs. Based on the risk assessments with multi‐parameters, it has been verified that long‐term transport and transformation of MPs in the deeper sediments could reduce the potential environmental and ecological risks. Source analysis indicates that anthropic activities and offshore operations were the primary sources of MPs deposited in cold seepage environments.

## 5 Experimental Section

### Abbreviation

PU; PA; PET; Polyoxymethylene (POM); PC; PE; PP; Polystyrene (PS); Polybutadiene (PBD); Polytetrafluoroethylene (PTFE); PVC; PSF; Polymethyl methacrylate (PMMA); AC; SI; FKM; and Phenol‐formaldehyde resin (PF).

### Sample Collection

Sediment cores were collected in the Haima cold seep, South China Sea, in May 2021. The Haima remotely operated vehicle (ROV) first dove and cruised along the seafloor. High‐definition videos were obtained to identify the environmental and ecological characteristics of the five diving locations. The detailed habitat situation depicted in Figure 1a revealed the non‐seepage areas, early seepage area, and middle seepage area with strong and weak methane flows. There was strong methane bubble ebullition with biomarkers of deep‐sea white clams and mussels in the sampling station of ROV1. Tube worms, deep‐sea white clams, mussels, large blocks of carbonates, and much lower flux methane were discovered in ROV2. Only microorganism mats and white clams were found in ROV3. ROV4 and ROV5 contained no biomarkers or geochemical markers. There were no special biomarkers of cold seepage in these two areas. Box coring was conducted at each dive location to acquire sediment cores. After returning to the scientific ship, several PVC cores were closed which were wrapped with tin foil sheets were inserted into the box corner to obtain sediment cores at the same diving locations with the same length.

### Sample Testing

One core was used for the MP extraction and identification. This core was stored at −20 °C until sent to the lab to extract and identify MPs. A combined test method was established to identify full‐scale (20–5000 µm) MPs, and this method was introduced step by step in Section S2.1, Supporting Information.

Another core was drilled to obtain sediment for DNA detection at 2 cm intervals, and these samples were wrapped in tinfoil and stored in the refrigerator (−80 °C) until DNA extraction. The DNA extraction procedure was explained in detail in Section S2.4, Supporting Information. After the DNA‐testing sample was retrieved, pore water was collected using a soil solution sampler of the rhizosphere on a scientific research ship. The pore water was sent to the lab for solution gas testing via gas chromatography (Trace1300/1310, Thermo Fisher, Waltham, MA, USA). The ion concentrations were measured via IC (Aquion, Thermo Fisher), inductively coupled plasma‐mass spectrometry (ICP‐MS; Thermo Fisher 7500a), and ICP‐optical emission spectrometry (ICP‒OES; Thermo Fisher 7000), which are introduced in Section S2.3, Supporting Information.

### Sediment Dating

Establishing sediment chronological sequence was an important means to restore and reconstruct past environmental features. Another core was cut into slices of 2 cm thickness each. A subsample of 5 g was extracted for age dating, and these samples were preserved without disturbance until sent to Yunnan University for dating analysis. In this work, the CRS age model of the ^210^Pb_bex_ activity decay and the peak value of ^137^Cs activity were used to establish and correct the age series of the sediment.^[^ ^68^ ^] 210^Pb dating method has a significant advantage in short‐time scale dating, which has an effective measurement range of 100–150 years. The measuring principle and procedure were introduced in detail in Section S2.2, Supporting Information.

### Risk Assessment for MPs

Four indicators including the PLI, PHI, PRI, and NPI were applied to assess the contamination of MPs in the deep‐sea area based on the following equations^[^ ^69^, ^70^ ^]^

PLI:

(1) $${\mathrm{CF}}_{i}=\frac{C_{i}}{C_{0}}$$

where CF*_i_* is the contamination factor of MPs at the sampling point *i*, expressing the ratio of the measured concentration (*C_i_*) to the background concentration (*C*_0_) of the MPs. The lowest abundance of the detected MPs was considered as the background concentration.

(2) $$\mathrm{PLI}=\sqrt{{\mathrm{CF}}_{i}}$$

(3) $${\mathrm{PLI}}_{r}=\sqrt[{n}]{{\mathrm{PLI}}_{1}\times {\mathrm{PLI}}_{2}\times \cdots {\mathrm{PLI}}_{n}}$$

where PLI*_r_* was classified into four categories. For example, <10 (low level of pollution), 10–20 (medium), 20–30 (high), and >30 (extremely high). *N* is the number of sampling points for ROV r in Equation (3).

PHI:

(4) $$\mathrm{PHI}=\sum_{n=1}^{n}P{}_{n}S{}_{n}$$

where PHI is the calculated polymer risk index; *P_n_* is the proportion of each polymer in each sample; and *S_n_* is the hazard score of the corresponding polymers in the MPs. PU, PA, PET, POM, PC, PE, PP, PS, PBD, PVC, PSF, PMMA, AC, SI, PKM, and PF had hazard score values of 1094, 50, 4, 871, 1177, 11, 1, 30, 6552, 10 001, 1, 1021, 10 599, 2, 2, and 750, respectively.^[^ ^71^ ^]^ The PHI values were classified into five categories: 0–1 was level I, 1–10 was level II, 10–100 was level III, 100–1000 was level IV, and >1000 was level V.

PRI:

(5) $$C_{f}=\frac{C_{i}}{C_{0}}$$

where *C_f_* is the enrichment coefficient of the MPs in the sampling point *i*.

(6) $${\mathrm{PRI}}_{i}=\sum_{n=1}^{n}\frac{P_{n}}{C_{i}}\times S_{n}$$

where PRI*_i_* represents the toxicity coefficient of the MPs. When the PRI values were <150, 150–300, 300–600, 600–1200, and >1200, the corresponding pollution levels were I, II, III, IV, and V, respectively.

NPI:

(7) $$\mathrm{NPI}=\sqrt{\frac{{\left(\frac{1}{n}\sum_{1}^{n}P_{i}\right)}^{2}+{\left(P_{i\max}\right)}^{2}}{2}}$$

where NPI is the Nemerow pollution index; NPI‐N is the Nemerow pollution index in terms of the number abundance; NPI‐M is the Nemerow pollution index in terms of the mass abundance; *P_i_* is the value of the pollution index for each sample point; and *P_i_*_max_ is the maximum value of the pollution index for each sample point.

(8) $$P_{i}=\frac{C_{i}}{C_{s}}$$

where *C_i_* is the measured value of the MPs at each sample point; *C_s_* is the standard reference value for the MPs, and 373 items kg^−1^ and 3465 mg kg^−1^ were selected in this study. The NPI values of ≤ 0.7, 0.7–1.0, 1.0–2.0, 2.0–3.0, and > 3.0 corresponded to the pollution levels of I, II, III, IV, and V, respectively.

The procedures of data calculation and statistical analysis were also described in Section S3, Supporting Information.

## Conflict of Interest

The authors declare no conflict of interest.

## Author Contributions

J.C.‐F. conceived and drafted the manuscript. C. R.‐L. and X.N.‐W. conducted the experiments. C. R.‐L., L.‐T., and Y.‐W. analyzed the data and generated the figures. L.W.‐S. collected the field samples. S.‐Z. and Z.F.‐Y. revised the manuscript.

## Supporting information

Supporting Information

Supporting Information

Supporting Information

Supporting Information

## Data Availability

The data that support the findings of this study are available from the corresponding author upon reasonable request.
