# Supplementary material for: Tracing the Century‐Long Evolution of Microplastics Deposition in a Cold Seep
Source: Adv Sci (Weinh). 2023 Feb 3;10(10):2206120. doi: 10.1002/advs.202206120 (PMC10074074; doi:10.1002/advs.202206120)
Supplement: Supplementary file 1 — Supporting Information [file ADVS-10-2206120-s003.pdf]

ADVANCED SCIENCE
Open Access

Supporting Information

for *Adv. Sci.*, DOI 10.1002/advs.202206120

Tracing the Century-Long Evolution of Microplastics Deposition in a Cold Seep

Jing-Chun Feng, Can-Rong Li, Li Tang, Xiao-Nan Wu, Yi Wang, Zhifeng Yang*, Weiyu Yuan, Liwei Sun, Weiqiang Hu and Si Zhang**

Supplementary Materials for

Tracing the century-long evolution of microplastics deposition in a cold seep

This file includes:

1. Geological information

2. Materials and methods

3. Data calculation and analysis

4. Figures S1 to S7

5. References

Other supplementary material for this manuscript includes the following:

Data S1 (MPs_identification&analysis_abun.xlsx)

Data S2 (Geochemical_con.xlsx)

Data S3 (Micro_gen.xlsx)

1. Geological information

This scientific investigation was conducted in the "Haima" cold seep area in May, 2021 by the Scientific Research Vessel "HAIYANG DIZHI LIUHAO". Geological and habitat characteristics were surveyed by the submersible remotely operated vehicle (ROV) "Haima". The water depth of each diving location was measured using single beam bathymetry (Kongsberg, EA600). Based on the information of latitude, longitude, and water depth, the geological map of Figure 1 was drawn using the software GMT V6.2. The seafloor relief data originated from http://dx.doi.org/10.1029/2019EA000658.

The development stages of the three methane seepage areas were based on the geological and habitat characteristics[1]. The complete development cause of the cold seep is shown in Figure S1. ROV1, ROV2, and ROV3 corresponded to the middle cold seep development stage with strong methane seeping (M&S seepage), the middle cold seep development stage with weak methane seeping (M&W seepage), and the early cold seep development stage (early seepage), respectively.

2. Materials and methods

2.1. Extraction and identification of the MPs

The method for extracting and identifying the MPs in this study included pretreatment, morphological observation, and composition identification[2,3]. Sizes from 20 to 500 μm and over 500 μm were recognized. Pretreatment primarily consisted of cleaning the organic materials and enriching the MPs in the sediment onto the membranes, which was the preparatory work for the size screening of MPs. Subsequently, morphological observations and composition identifications were used to identify the sizes of the MPs. Large-size plastics with the scale of over 500 μm were first removed. Next, MPs from 20 to 500 μm were identified. The specific steps and equipment used in the each process are listed in Figure S2.

2.2. Sediment dating

In this study, two isotope dating systems were used to obtain more accurate and reliable sediment age information. The measurement range of ^{210}Pb dating method is 100—150 years. The natural radioactive lead isotope ^{210}Pb is an alpha decay of the intermediate ^{222}Rn (3.8d half-life) in the decay process of ^{226}Ra (series ^{238}U with a half-life of 1622a), which has a

half-life of 22.3a. ^{222}Rn is released from environmental media such as rock surface and soil particles into the lower atmosphere. Under the influence of local differences in air transport and escape ratio, ^{222}Rn in the lower atmosphere also changes. The ^{222}Rn in the atmosphere comes mainly from the land surface. The ^{210}Pb in the atmosphere enters the water through dry and wet sedimentation, collects in the sediment, adsorbs on the suspended particles, and deposits in the deep sea bottom with the suspended matter year by year to form a self-closed system. The ^{210}Pb accumulated in sediments is usually referred to as excess ^{210}Pb ($^{210}Pb_{bex}$) because it does not coexist and balance with its parent ^{226}Ra. By analyzing the $^{210}Pb_{bex}$ specific activity of sediment core samples at different depths, the deposition rate or the deposition age of a particular layer can be calculated.

The CRS age model of the $^{210}Pb_{bex}$ activity decay and the peak value of ^{137}Cs activity were used in this study to establish and correct age series of the sediment [4]. Four steps were generally included. First, the freeze-dried samples were ground and placed into the tubes of the dating sample. Second, the sample surface was compacted to approximately 4 cm to flatten it. Then approximately 1 cm of epoxy AB adhesive was injected Third, the $^{210}Pb_{bex}$ activity of the sample was measured a week later using a high-purity germanium detector, a digital spectrometer, and a multi-channel analysis system (Canberra company, US). Fourth, the data were analyzed to establish the age series of the entire borehole. ^{210}Pb is the most common isotope used to date the sedimentary core. At least one stratigraphic tracer had to be used during the dating test. ^{137}Cs was used in the experimental process.

2.3. Geochemical environmental factors testing

2.3.1. Concentration of nutrients

The concentration of a nutrient in a water sample was determined by measuring its absorbance and comparing it with the standard curve of absorbance [5]. The measuring process including five steps based on part four of the national standard GB/T12763.4(2007) [6]. First, the pore water samples were removed from the refrigerator at −20°C and then thawed in the refrigerator at 4°C. Second, the samples were transferred using a 0.45 μm disposable needle filter[7]. Third, 1 mL of filtrate was removed, and 9 mL of ultra-pure water was added to obtain

the pore water that was diluted 10 times. Then, the diluted samples were sent to the sampling ring for detection using an automatic sampler and a peristaltic pump. Finally, the data were compared and analyzed with the standard sample to obtain the concentration of the nutrient. Five nutrients of phosphate, nitrite, ammonium salt, nitrate, and silicate were measured in this study. The equations used for the calculation were as follows.

Phosphate and nitrite:

$$C = \frac{(\bar{A}_w - A_b) - a}{b} \tag{1}$$

Ammonium salt:

$$C = \frac{\bar{A}_w - A_b - X \bullet \bar{A}_{NO_2^- - N} - a}{b} \tag{2}$$

Nitrate:

$$C = \frac{\bar{A}_w - A_b - X \bullet \bar{A}_{NO_2^- - N} - a}{b} \tag{3}$$

Silicate:

1） salinity(S) > 7

$$C = \frac{(\bar{A}_w - A_b)(1.05 + 0.001S) - a}{b} \tag{4}$$

2） salinity(S) ≤ 7

$$C = \frac{\left(\bar{A}_w - A_b\right)(1.0 + 0.008S) - a}{b} \tag{5}$$

Here C is the concentration of phosphorus [P] in phosphate, nitrogen [N] in nitrite, nitrogen [N] in ammonium, nitrogen [N] in nitrate, and silicon [Si] in silicate. Their units are μmol/L. $\bar{A}_w$ is the average absorbance value of a water sample; A_b is the blank absorbance value; a is the intercept of the standard working curve; b is slope of the standard working curve; $\bar{A}_{NO_2^- - N}$ is the average absorbance value measured during the "nitrite determination" (reagent blank has been deducted); and X is length ratio of the colorimetric cell. The value of 0.4 was

used for the "nitrate determination" and the "nitrite determination" according to the conditions in the national standard. S is salinity.

2.3.2. Total organic carbon and dissolved inorganic carbon

The total organic carbon analyzer (TOC-L CPH) was used to determine the content of the total organic carbon (TOC) and dissolved inorganic carbon (DIC) in the sediment [6]. Samples were be pretreated prior to determining the TOC and DIC contents. First, the sediment samples were removed from the −20°C refrigerator and thawed in the 4°C refrigerator. Second, approximately 3 g of each sample from each layer of sediment was removed and packed with tinfoil. Third, they were dried in a freeze dryer to remove the moisture. The freeze-dried samples were then ground with a mortar and sieved with a 200-mesh stainless steel screening. After finishing the above steps, the samples were sent to the TOC analyzer for testing. The process for the TOC determination included three steps. First, the samples were placed in the TOC combustion tube, where the samples were fully burned to generate CO_2. Second, the CO_2 was sent to the non-dispersive infrared (NDIR) gas detector after dehydration and dehalogenation occurred in the instrument. Finally, the content of TOC in the samples were calculated by calculating the peak area of CO_2.

Similarly, when the content of DIC was investigated, the samples were first sent into the DIC sampling tube and reacted with hydrochloric acid. Then the DIC in the samples were converted into CO_2 that could be detected by the NDIR gas detector. Finally, the content of DIC was also calculated according to the peak area of CO_2.

2.3.3. Metal and ionic concentrations

In this study, inductively coupled plasma-mass spectrometry (ICP-MS, iCAP-RQ) and an inductively coupled plasma-optical emission spectrometer (ICP-OES, iCAP-7000 SERIES) were used to determine the concentrations of the metal elements of ferrum [Fe], manganese [Mn], hydrargyrum [Hg], and sulfur [S] in the pore water. Ion chromatography (IC) was used to determine the anion of sulfate [SO_4^{2-}]. During the determination of the metal and sulfur elements, the water sample was filtered through a 0.22 μm membrane to remove impurities or sediment, and then the dilution factor was adjusted according to the ionic concentration of the

sample and then diluted with a mixture of 2%HNO_3 and 0.5%HCl acid and tested on the machine. Similarly, during the process of determining the anion, the water samples were diluted 1000 times with the NaOH solution (pH = 12). Next, the impurities or precipitates were removed using a 0.22 μm filter membrane. Finally, the anion was determined using the post-column derivatization ion chromatograph (AQ-1200).

2.3.4. Methane concentration

The collected pore water was placed into an anaerobic bottle, and mercury chloride was added and stored at 4°C. The gas in the upper portion of the collected bottle was detected using a modular gas chromatograph (Thermo Scientific TRACE 1300, USA).

2.4. DNA sequencing

The total genomes were extracted. For each sample, total genomic DNA was extracted three times for technical replication. For each technique replicated, about 2 g of sediment was used to extract total genomic DNA after swirl mixing. NucleoSpin soil kit (Machey-Nagel, Germany) was used to extract total genomic DNA according to manufacturer's instructions. Gel electrophoresis and a qubit fluorometer were utilized to evaluate the quality and purity of the extracted DNA. The qualified genes were amplified by PCR and purified by Agcourt AMPure XP beads (Beckman Coulter, USA). Then the database was established. The established database was evaluated with the Agilent 2100 BioAnalyzer (Agilent, USA). Sequencing was performed on the Illumina HiSeq 2500 PE300 platform after passing the database. BGI Genomics (Shenzhen, China) was commissioned to conduct the DNA extraction, PCR amplification, and sequencing of the 16S rRNA genes in this study.

The primer set designed in this study was 338F (5'-ACTCCTACGGGAGgCAGCAGcag-3'). 806R (5'-GGACTachVGGgTWTCTAat-3') was used to obtain the V3–V4 variable regions of the bacterial 16S rRNA gene[8]. BGI Genomics processed the raw data. High-quality sequences were generated. VSEARCH V2.7.0 was used to dispose the sequence and analyze the step [9]. The command of uchime_ref was based on the SILVA 132 database [10] that was used to detect the operation taxonomic units (out). QIIIME V.1.9.0 [11] was used to classify and allocate the OTU sequences. All raw data were submitted

in NCBI Sequence Read Archive (accession code PRJNA845826).

3. Data calculation and analysis

3.1. Statistical quantification of the MPs

The abundance of MPs is an important indicator to determine the degree of MP pollution. The abundance was calculated from the perspective of the number and mass in this study. The equation utilized were as follows.

Number abundance:

$$NA = \frac{Q_{>500} + Q_{20-500}}{M}, \tag{6}$$

where NA is the number abundance of MPs, and its units are items/kg; $Q_{>500}$ is the number of MPs with sizes over 500 μm; Q_{20-500} is the number of MPs with sizes from 20 to 500 μm; and M is quality of the corresponding sediment samples, and its unit is kg.

Mass abundance:

$$MA = \frac{1000 \times \sum_{i}^{j} \rho_x v_x}{M}, \tag{7}$$

where MA is the mass abundance of MPs, and its unit is mg/kg. ρ_x is the density of the corresponding polymer, and its unit is g/cm^3; V_x is the sum of volumes of the corresponding polymer, and its unit is cm^3; x is the different polymer types $i \cdots j$; and M is the quality of sediment samples at the corresponding horizons, and its unit is kg.

3.2. Morphological quantification of MPs

The morphological characteristics of color, shape, particle size, and the corresponding polymer in the sample were recorded during the observation of MPs in the inverted fluorescence microscope. The abundance of the corresponding morphological characteristics was calculated using the following equations:

Color:

$$CA_y = \frac{Q_y}{Q} \times NA, \tag{8}$$

where CA_y is the abundance of the corresponding color, and its unit is items/kg; Q_y is the number of the corresponding colors, and its unit is items; Q is the number of MPs observed

in the fluorescence microscope, and its unit is items; and NA is the number abundance of MPs, and its unit is items/kg.

Shape:

$$SHA_t = \frac{Q_t}{Q} \times NA, \tag{9}$$

where SHA_t is the abundance of the corresponding shapes, and its unit is items/kg; Q_t is the number of the corresponding shapes, and its unit is items; Q is the number of MPs observed in the fluorescence microscope, and its unit is items; and NA is the number abundance of MPs, and its unit is items/kg.

Particle size:

$$SA_n = \frac{Q_n}{Q} \times NA, \tag{10}$$

where SA_n is the abundance of the corresponding particle size, and its unit is items/kg; and Q_n is the number of particles of different size grades, and its unit is items. In this work, the MP size grade was divided into six levels. Q is the sum of number of MPs detected by LDIR with sizes from 20 to 500 μm and according to the FTIR with sizes greater than 500 μm, and its unit is items; and NA is the number abundance of MPs, and its unit is items/kg.

Polymer:

$$PA_n = \frac{Q_f}{Q} \times NA, \tag{11}$$

where PA_n is the abundance of the corresponding polymer, and its unit is items/kg; Q_f is the number of the corresponding polymers in the sample, and its unit is items; Q is the sum of the number of MPs detected by the LDIR with sizes from 20 to 500 μm and by FTIR with sizes greater than 500 μm, and its unit is items; and NA is the number abundance of MPs, and its unit is items/kg.

3.3. Burial rate

The burial rate was calculated as follows:

$$BR = \frac{M}{S \times A}, \tag{12}$$

where *BR* is the burial rate; *M* is mass abundance of the sedimentary layers of the corresponding age; *A* is the age of the sedimentary layers; and *S* is the area of the PVC pipe based on its diameter.

The following equation was used to perform the normalization. The maximum and minimum values (0 and 1) were removed after the burial rate was normalized. Then SPSS 28.0 was used for the exponential fitting.

$$X_{norm} = \frac{X - X_{\min}}{X_{\max} - X_{\min}}, \quad (13)$$

where X_{norm} is the normalized standard values for the 2 cm of the sediment layer; *X* is the corresponding deposition rate; X_{max} is maximum value of the deposition rate at a certain station; and X_{min} is minimum value of the deposition rate at a certain station.

Considering the uncertainty of the data testing, the burial rate from approximately the 1970s was chosen as the average value for the ROV. The detailed data are provided in Data S1 (NumMPs_identification&analysis.xlsx).

3.4. Statistical analysis

Figure 1c, d was drawn using R language version 4.1.3. The correlation between the non-S1 and the GPP as well as the non-S2 and the GPP was performed based on the Spearman rank correlation coefficient in the R Packet Psych. Figure 2a, b was drawn by the R language Vegan package. The principal component analysis (PCoA) community difference analysis was performed based on the Bray-Curtis distance and the significance test of the permutation multiple variance analysis (PERMANOVA). Figure 3c–e was drawn by R language. Figure 4a was analyzed in R language using the non-metric multidimensional scaling analysis (NMDS). The Spearman correlation coefficient in R language was used to draw the chart on the left side of Figure 4e. Figure S4f was performed by the ggpairs function of the R package GGally in R language. Figure 5a was drawn by the R package Vegan in R language.

The network analysis in Figure 5c, d was used by the R package Psych in R language based on the Spearman correlation coefficient. In this, the point files, edge files, and

correlation coefficient files were imported into Cytoscape 3.8 software, and these charts were output. The results of the function prediction data of the KEGG KO library were based on the MicFunPred database. The core genes predicted at the genus level in the given nucleotide sequence were used, and the enrichment method of the minimum path was considered. The function of the bacterial community was estimated and predicted using a conservative channel prediction. The function prediction files based on the KEGG, EC, COG, and Pfam were obtained and compared in the KEGG KO database to obtain the graphs. The linear discriminant analysis effect size (LEFSe) was conducted using R packet Coin and MASS packet in R language. The linear discriminant analysis (LDA) model was used to reduce and evaluate the influence of bacteria with significant differences in each layer of the sediment flora, which is shown by the LDA score (Fig. 6b).`

4. Figures S1 to S7

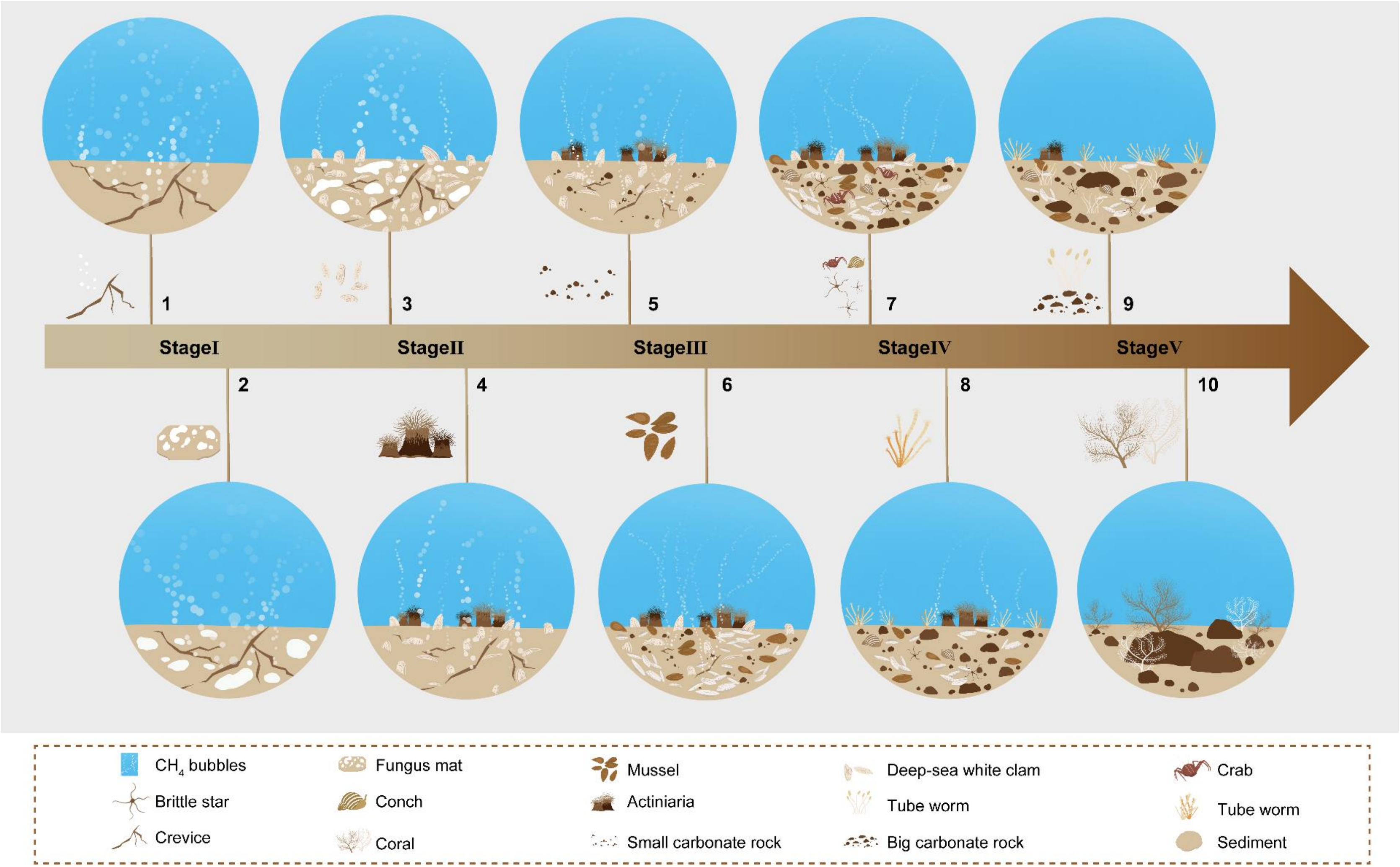

Figure S1. Development stages of the cold seep.

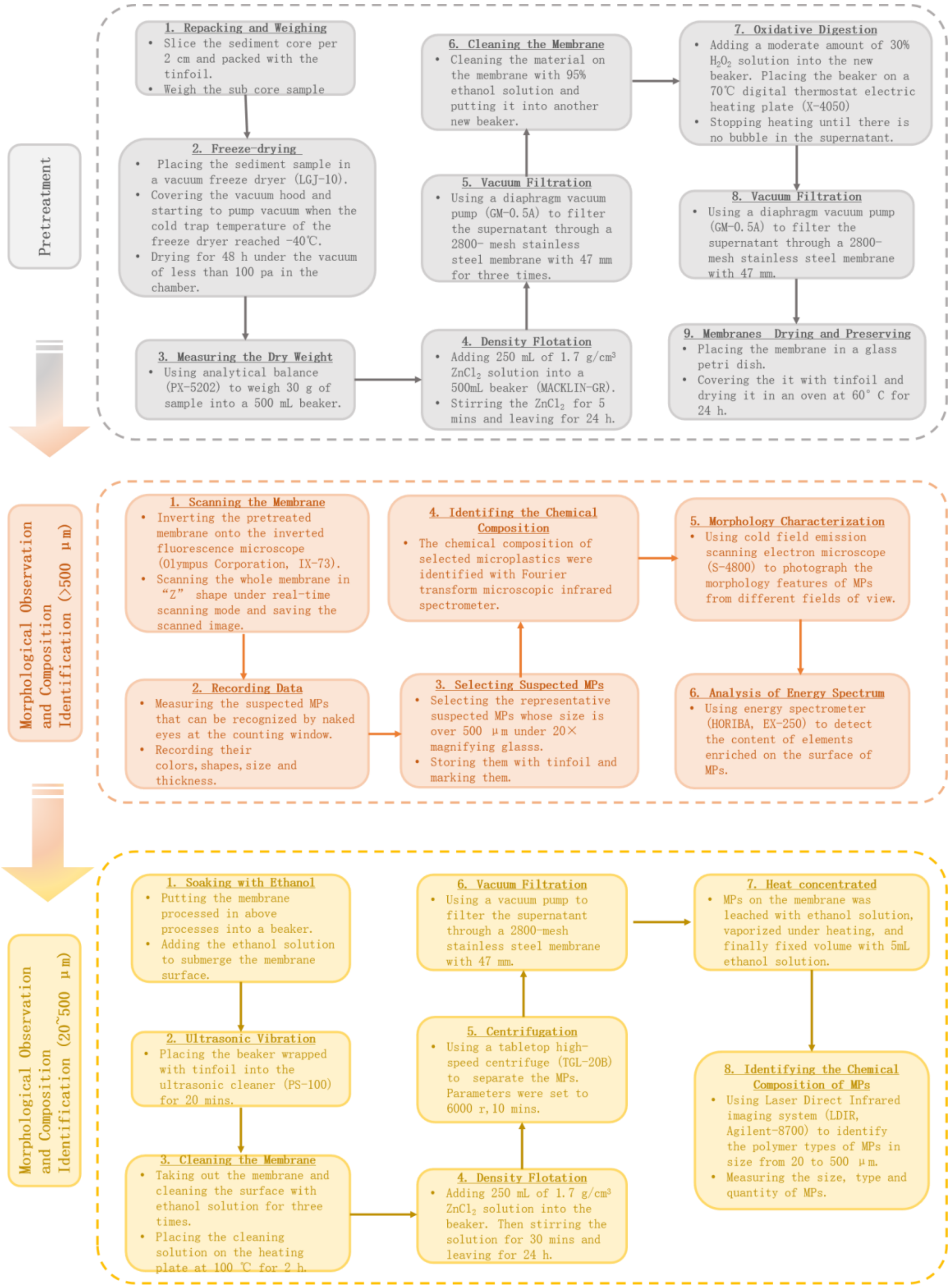

Figure S2. Processes for the full-scale (20 μm–5 mm) extraction and identification of the MPs.

This work adopts a full-size extraction and detection method of microplastics in the sediments of deep-sea cold spring described in the original patent. The specific content includes the pretreatment of deep sea cold spring sediments to obtain the purified microplastic solution. The microplastic stainless

steel purified membrane with size > 5um was obtained by vacuum filtration. Then, inverted fluorescence microscope was used to perform Z-scanning on the purified filter membrane to detect the morphological data of microplastics > 5um in the sediments of deep-sea cold spring area. We divided the size range of microplastics into > 500um and 20-500um, and developed a relatively optimized detection scheme according to the different sizes. For MPs of > 500um, microplastics of > 500um will be selected from stainless steel purified filter under a magnifying glass (× 20x), then their components will be determined under Fourier transform microinfrared spectrometer and polymer energy spectrum will be taken under a cold field scanning electron microscope to obtain the surface microscopic properties. For MPs of 20~500um, extraction and centrifugation will be performed on the purified filter membrane to obtain the microplastic extraction and purification solution, which will be filtered onto the new stainless steel membrane under vacuum filtration. After drying at room temperature, MPs will be concentrated on the membrane with ethanol solution, and then dripped onto the silver-plated glass sheet. The components were determined by Agilent LDIR laser infrared imaging system.

Different from the detection of microplastics in this work, currently common detection methods of microplastics in sediments in water environment adopt glass fiber filter membrane as the main filtration membrane, the method of dissolution before flotation and the pretreatment method of grinding. These methods have some shortcomings, such as introducing translucent fibers to detect major interference sources and floating fluff-like impurities in sediments to the supreme clear liquid. Resulting in difficult purification problems and > 1mm microplastics may be ground during grinding.

In the whole MPs detection scheme, the simple digestion method of density flotation followed by membrane digestion is adopted to greatly reduce the usage of hydrogen peroxide and improve the experimental efficiency. Moreover, the step of centrifugal filtration is added to greatly reduce the influence of particle background generated by zinc chloride and ensure that the qualitative microplastics are within the allowable error range during morphological observation. In addition, this work did not use the common glass fiber filter membrane, but used inorganic membrane with strong mechanical strength, which makes it easy to observe microplastics under the microscope background. At the same time, there is no interference caused by organic components, which reduces the experimental influence caused by cellulose escape during subsequent ultrasonic, soaking and shock

after the use of glass fiber filter membrane. Moreover, the identification effect of Fourier transform micro-infrared spectrometer and laser infrared instrument is enhanced and convenient for subsequent plastic recycling experiments. It is a set of one-stop detection method for microplastics covering 20um.

Figure 2-e shows the ecological risk assessment of sediment microplastics in five stations. The risk assessment indexes of pollution load index (PLI), polymer risk assessment index (PHI), potential ecological risk index (RI) and Nemerow pollution index (Newerow) are adopted. At the same time, Nemerow pollution index was used to verify and calibrate the harm caused by the quantity and quality of microplastics respectively, and analyze the risk and harm of samples from a multi-dimensional perspective. In Figure 2-e, the values obtained from all risk assessment methods are treated with risk level normalization, so that all values can be uniformly assessed according to the five risk levels. Conclusion Analysis: PLI→ROV1 and ROV3 have the highest pollution load index, and ROV2 has the lowest pollution load index, indicating that in the early stage of methane ecozone, plastics have shown signs of fragmentation, leading to a large difference in background concentration, but because the background value itself is too high, this difference is not significant. In the middle stage of methane ecozone, microbial life activities were intensified, microplastics were degraded more, and the overall difference decreased, as shown by the decrease of PLI value of ROV2. Since the background value of the pollution load index is the lowest value of the sample itself, this assessment method will lead to certain risk grade assessment errors, but the relative trend of this background value can be used to mine the conclusion. If the calculation is based on the safe concentration of microplastics in seawater/reef abundance, the risk level of this method must be very high. PHI→ The polymer types of the 5 stations are similar, so the calculated values of all stations are high. For the whole site, the PHI contribution value of PC to microplastics is the highest only in the surface layer, followed by acrylic ester and PU. RI→ This index is calculated according to the value of PLI and PHI, so the size of RI depends on the above two indices. The ROV2 value is the smallest, indicating that the methane ecological zone has a stronger ability to degrade plastics in the middle stage than other stations. The indices Newerow-ant and Newerow-ali→ were calculated with the safety concentration of microplastics in sediments converted from the literature as the background value. From the perspective of quantity abundance, the risk levels of all stations exceeded the maximum, and the index of methane ecozone was smaller than

that of non-methane ecozone, and the pollution degree was also smaller. From the perspective of mass abundance, the index of total site decreased with the increase of sediment depth. It is not possible to generalize the risks and hazards of MPs only by assessing their abundance. Therefore, multi-angle index method should be adopted to comprehensively evaluate the ecological risk assessment of MPs.

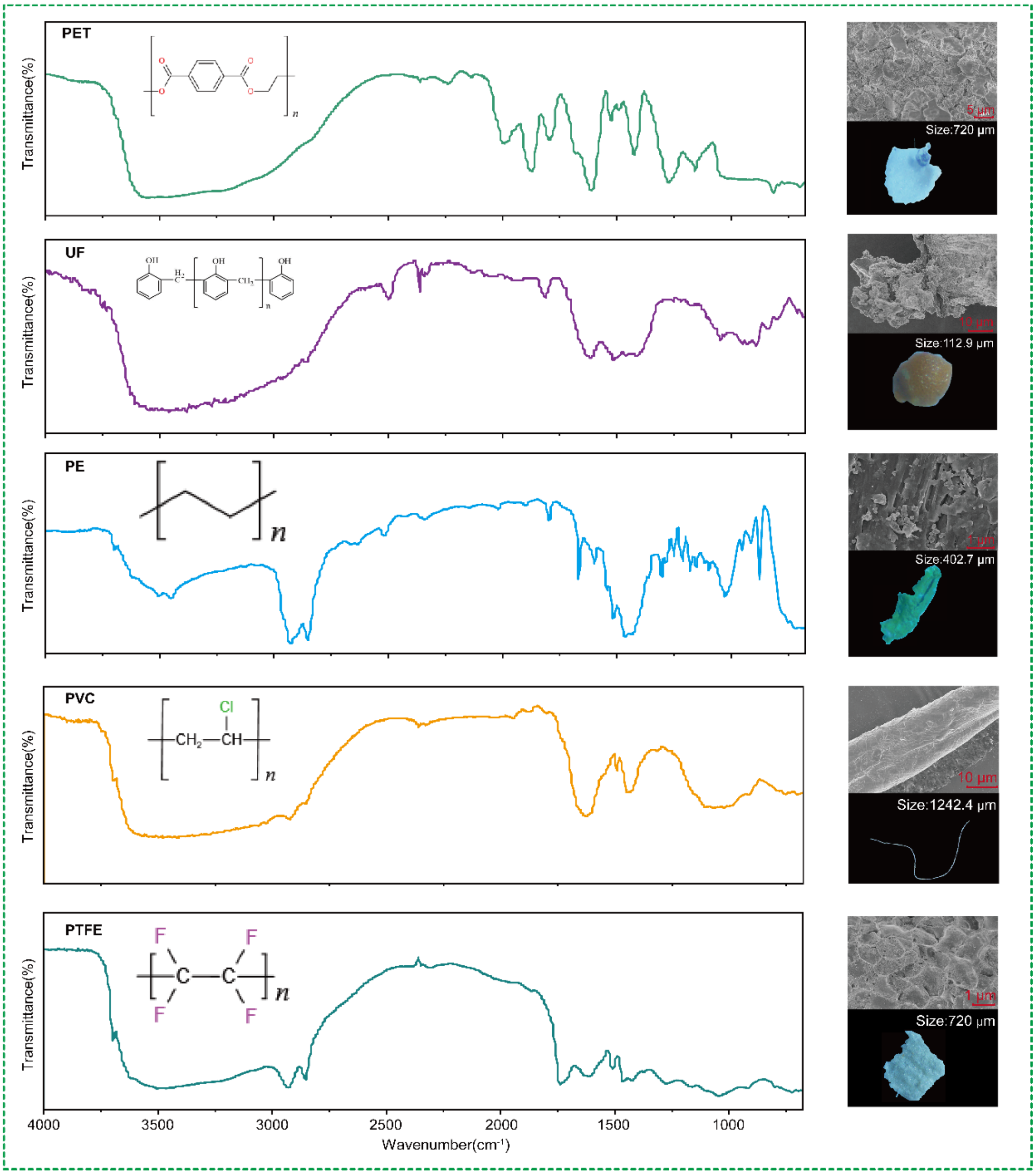
PET
UF
PE
PVC
PTFE
Transmittance(%)
Size:720 μm
Size:112.9 μm
Size:402.7 μm
Size:1242.4 μm
10 μm
4000
3500
3000
2500
2000
1500
1000
Wavenumber(cm⁻¹)

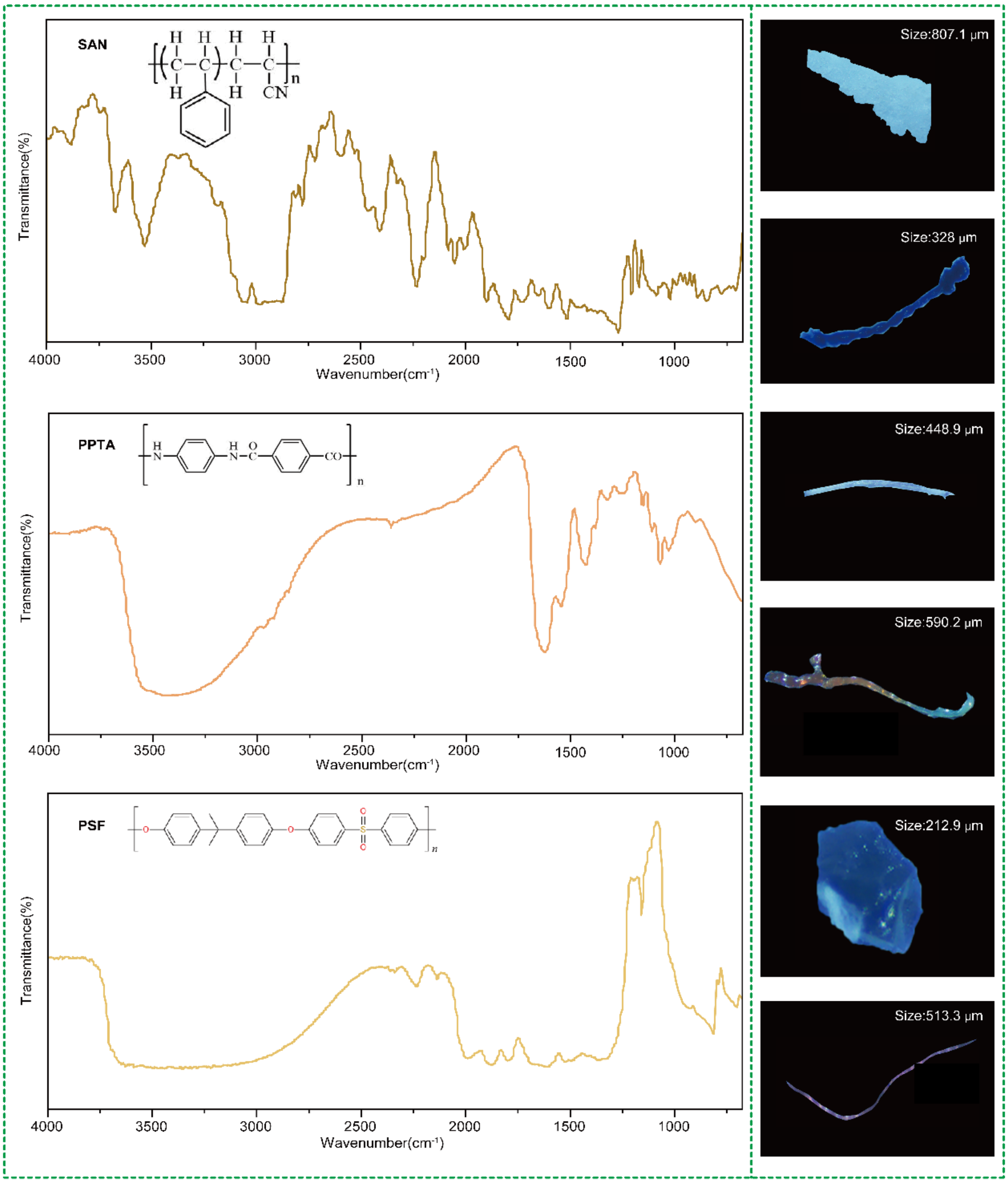

Figure S3. Images and spectrogram of the large-scale MPs (>500 μm).

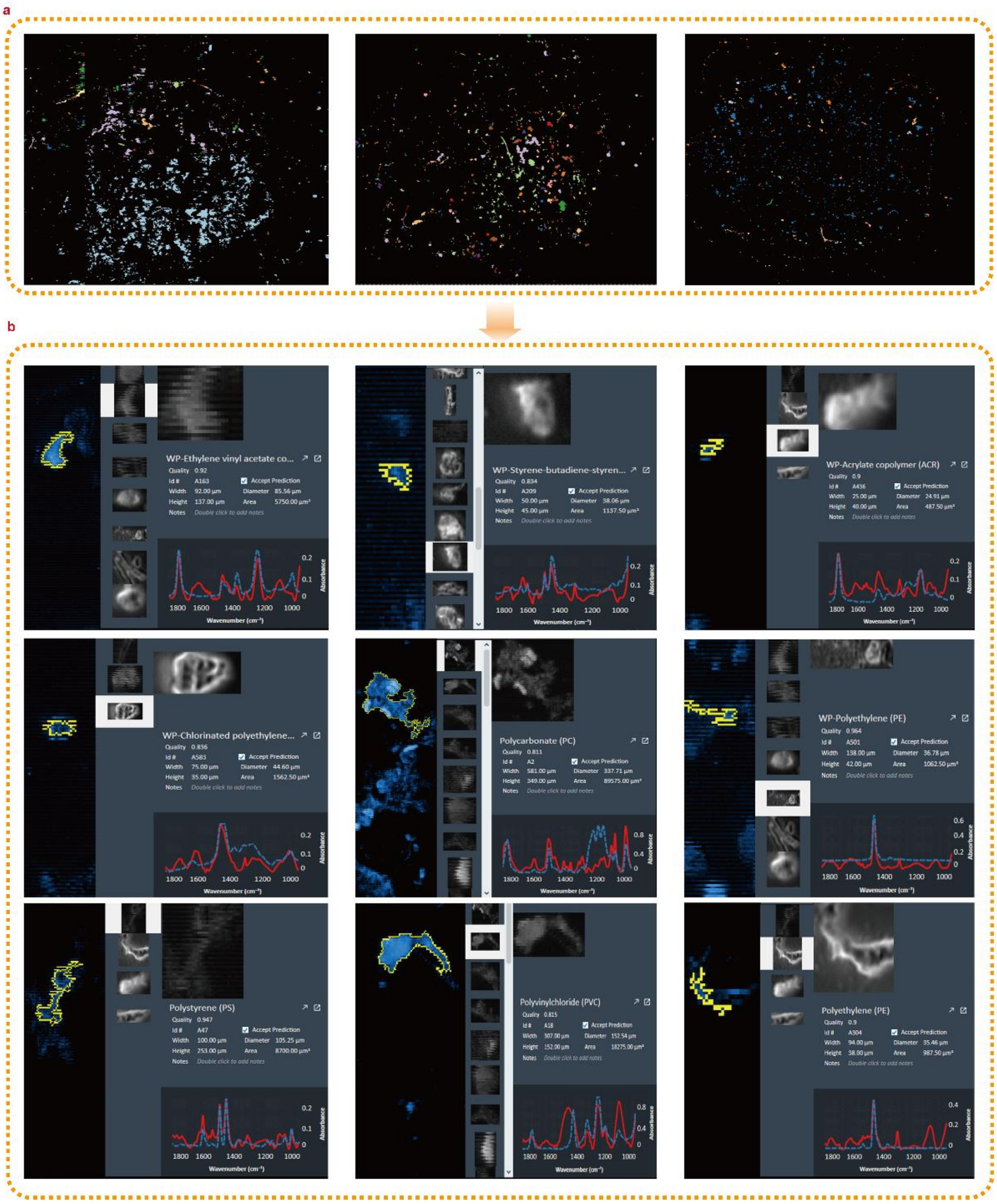
a
b
WP-Ethylene vinyl acetate co...
WP-Styrene-butadiene-styren...
WP-Acrylate copolymer (ACR)
WP-Chlorinated polyethylene...
Polycarbonate (PC)
WP-Polyethylene (PE)
Polystyrene (PS)
Polyvinylchloride (PVC)
Polyethylene (PE)
Quality
Id #
Width
Height
Notes
Accept Prediction
Diameter
Area
Double click to add notes
Absorbance
Wavenumber (cm⁻¹)

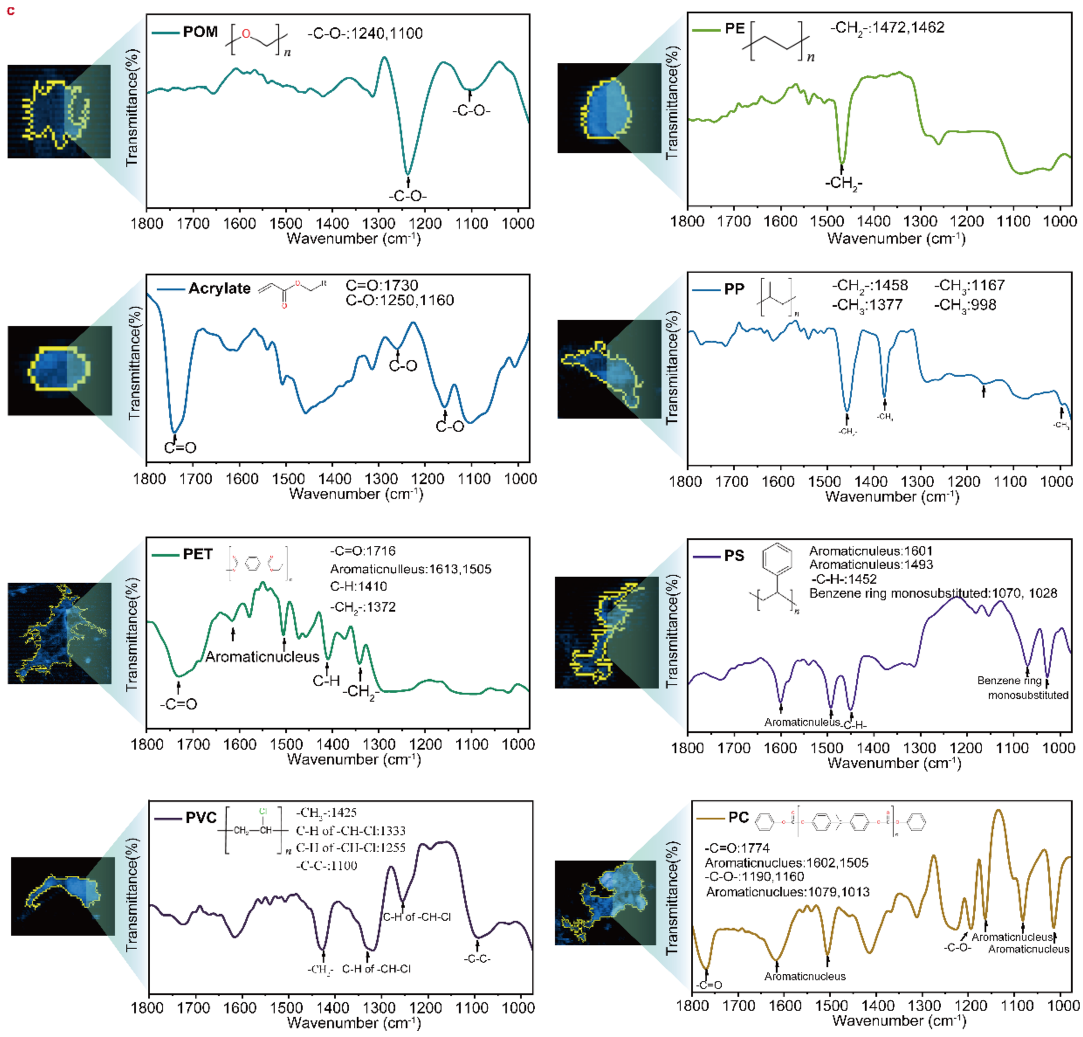

Figure S4. Morphological characteristics the small-scale MPs (20–500 μm). (a) Overview of the species distribution of the identified particles in the LDIR scans. The different types of MPs or impurities are indicated as different colors. (b) Spectrograms with a higher signal-to-noise ratio obtained by comparing the detected MPs with the standard spectrum library using the clarity microplastic detection module. The accepted match degree was higher than 65%. (c) Laser infrared spectrogram of the granular or fibrous MPs.

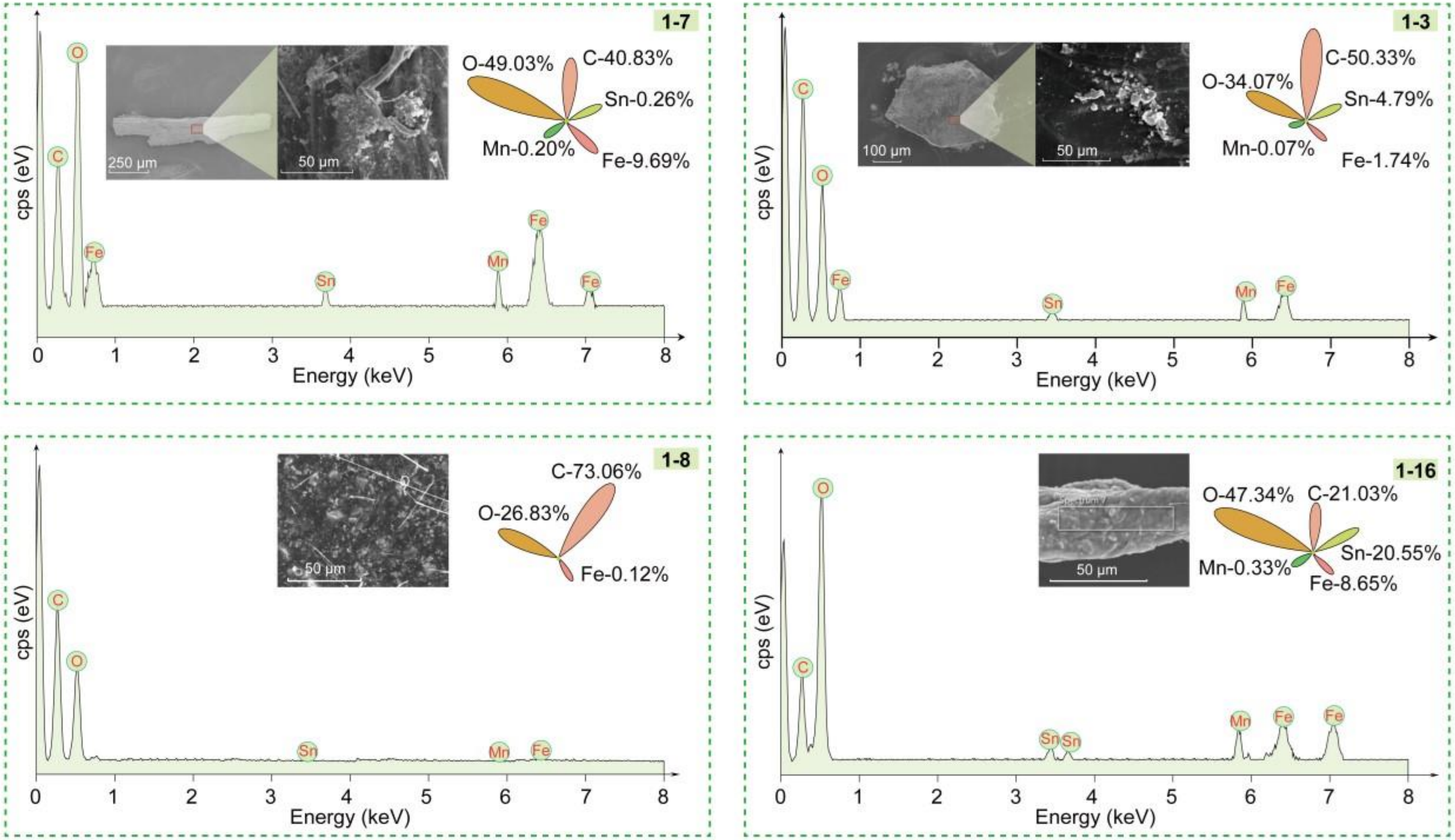

Figure S5. SEM-EDS analysis results of the MPs in each layer of sediment depth at the M&S seepage

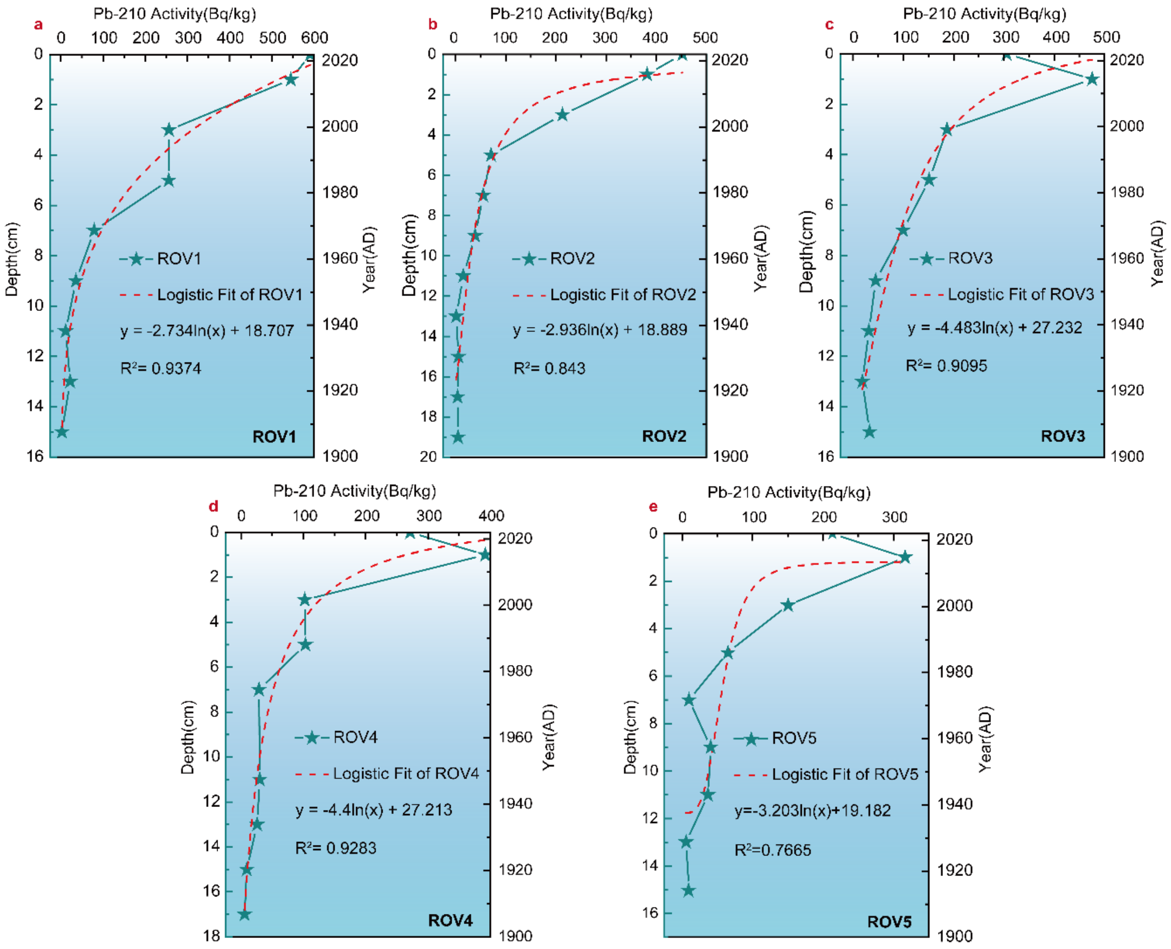

Figure S6. CRS dating model corrected by ^{137}Cs for the ^{210}Pb activity.

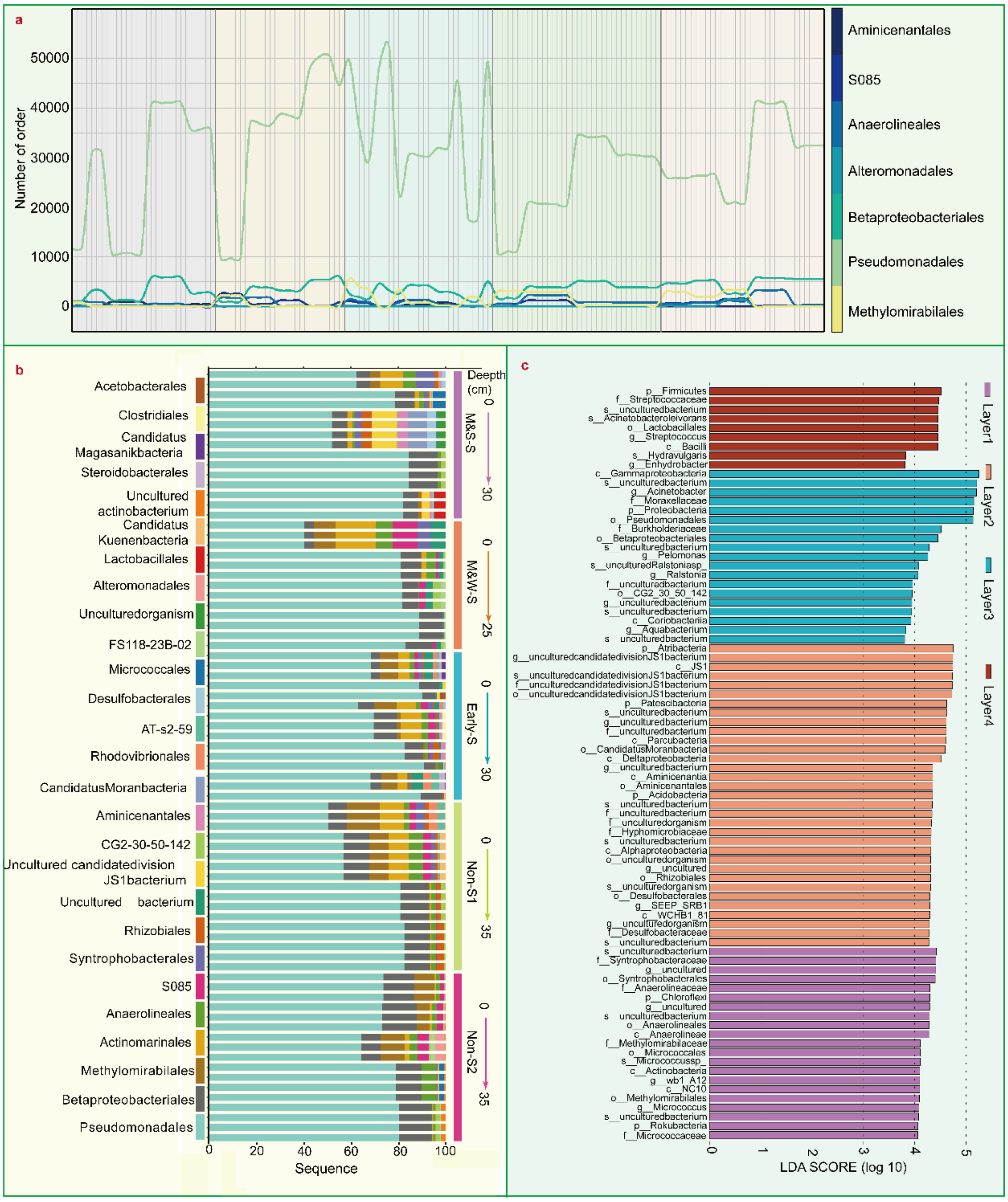

Figure S7. Bacterial abundance and LDA test results. (a) *Aminicenantales*, *S085*, *Anaerolineales*, *Alteromonadales*, *Betaproteobacteriales*, *Pseudomonadales*, and *Methylomirabilales* were selected from the OTUs. The absolute abundance was sorted by the station position and depth. (b) The bacterial composition distribution map sorted by the station depth after filtering out bacteria with relative abundances of less than 1% and classifying them by order. (c) The sediment mud column was divided into four layers with equal distances, and the weakly correlated populations less than 1% were filtered out. The LDA score in this figure was generally >4, and the model had a good interpretation effect. The missing sediment microflora in this study was obtained by fitting the data measured before and after.

5. References

1 Bowden, D. A. *et al.* Cold seep epifaunal communities on the Hikurangi margin, New Zealand: composition, succession, and vulnerability to human activities. *PLoS One* **8**, e76869, doi:10.1371/journal.pone.0076869 (2013).

2 Liebezeit, G. & Dubaish, F. Microplastics in Beaches of the East Frisian Islands Spiekeroog and Kachelotplate. *Bulletin of Environmental Contamination and Toxicology* **89**, 213-217, doi:10.1007/s00128-012-0642-7 (2012).

3 Zhang, D. *et al.* Microplastic pollution in deep-sea sediments and organisms of the Western Pacific Ocean. *Environmental Pollution* **259**, doi:10.1016/j.envpol.2020.113948 (2020).

4 Torres, F. G. & De-la-Torre, G. E. Historical microplastic records in marine sediments: Current progress and methodological evaluation. *Regional Studies in Marine Science* **46**, doi:10.1016/j.rsma.2021.101868 (2021).

5 Li, K. Methane preprocessing device of methane generator set comprises methane preprocessing tank connected to methane tank, desulfuration tank connected with methane preprocessing tank, and methane drying system connected to methane generator set. CN202576371-U.

6 GB/T12763.4(2007). *The specification for marine monitoring-Part 4: Seawater annalysis*.

7 Zhang, L. *et al.* Pore water nutrient characteristics and the fluxes across the sediment in the Pearl River estuary and adjacent waters, China. *Estuarine Coastal and Shelf Science* **133**, 182-192, doi:10.1016/j.ecss.2013.08.028 (2013).

8 Mori, H. *et al.* Design and Experimental Application of a Novel Non-Degenerate Universal Primer Set that Amplifies Prokaryotic 16S rRNA Genes with a Low Possibility to Amplify Eukaryotic rRNA Genes. *DNA Research* **21**, 217-227, doi:10.1093/dnares/dst052 (2014).

9 Kong, J. *et al.* Contrasting Community Assembly Mechanisms Underlie Similar Biogeographic Patterns of Surface Microbiota in the Tropical North Pacific Ocean.

Microbiology Spectrum **10**, doi:10.1128/spectrum.00798-21 (2022).

10 Yilmaz, P. *et al.* The SILVA and "All-species Living Tree Project (LTP)" taxonomic frameworks. *Nucleic Acids Research* **42**, D643-D648, doi:10.1093/nar/gkt1209 (2014).

11 Caporaso, J. G. *et al.* QIIME allows analysis of high-throughput community sequencing data. *Nature Methods* **7**, 335-336, doi:10.1038/nmeth.f.303 (2010).

12 Guo, Z. *et al.* Global meta-analysis of microplastic contamination in reservoirs with a novel framework. *Water Res* **207**, 117828, doi:10.1016/j.watres.2021.117828 (2021).

13 Polechońska, L., Klink, A., Dambiec, M. & Rudecki, A. Evaluation of Ceratophyllum demersum as the accumulative bioindicator for trace metals. *Ecological Indicators* **93**, 274-281, doi:10.1016/j.ecolind.2018.05.020 (2018).

14 Lithner, D., Larsson, A. & Dave, G. Environmental and health hazard ranking and assessment of plastic polymers based on chemical composition. Science of the Total Environment 409, 3309-3324, doi:10.1016/j.scitotenv.2011.04.038 (2011).
